# Characterization of a novel lncRNA RP3-340N1.2 and its association with a miR-4650-5p/SHC1-related regulatory network in lung adenocarcinoma

**DOI:** 10.1371/journal.pone.0353744

**Published:** 2026-07-16

**Authors:** Fang Chen, Yan Yan, Wenting Yang, Zhilin Li, Daying Wang, Jianqiao Huang, Chaozhou Wan, Tingting An, Li Tong, Maoxia Ran, Yaqiong Dong, Yunfen Chen

**Affiliations:** 1 Guizhou Medical University, Guiyang, Guizhou, P.R. China; 2 Guizhou Provincial Staff Hospital, Guiyang, Guizhou, P.R. China; 3 Guiqian International Hospital, Guiyang, Guizhou, P.R. China; 4 Affiliated Hospital of Guizhou Medical University, Guiyang, Guizhou, P.R. China; Xiangya Hospital Central South University, CHINA

## Abstract

Long noncoding RNAs (lncRNAs) are increasingly recognized as regulators of cancer-related biological processes. However, the functional significance of many lncRNAs in lung adenocarcinoma (LUAD) remains incompletely understood. In this study, we identified RP3-340N1.2 as an upregulated lncRNA in LUAD through analyses of public transcriptomic datasets, which was further confirmed by quantitative real-time PCR in LUAD cell lines. Functional assays demonstrated that knockdown of RP3-340N1.2 was associated with reduced proliferation, migration, invasion, and clonogenic growth of LUAD cells in vitro. In addition, suppression of RP3-340N1.2 attenuated tumor growth in a xenograft model. Bioinformatic analysis using the LncBase Experimental v3 database identified hsa-miR-4650-5p as a potential interacting microRNA of RP3-340N1.2. This interaction was further examined by dual-luciferase reporter and RNA immunoprecipitation assays. Functional experiments additionally showed that miR-4650-5p overexpression was associated with reduced proliferative and migratory capacities in LUAD cells. Among the predicted downstream targets of miR-4650-5p, SHC1 was selected for further investigation. Alterations in RP3-340N1.2 or miR-4650-5p expression were accompanied by corresponding changes in SHC1 expression and ERK1/2 phosphorylation. Furthermore, rescue experiments demonstrated that SHC1 knockdown largely reversed RP3-340N1.2-associated cellular phenotypes, supporting the functional involvement of SHC1 within this regulatory framework. Collectively, these findings indicate that RP3-340N1.2 is aberrantly expressed in LUAD and may participate in tumor-associated cellular behaviors through a miR-4650-5p/SHC1-related regulatory mechanism. This study provides preliminary evidence supporting the potential relevance of RP3-340N1.2 in LUAD and offers additional insight into lncRNA-associated regulatory networks in this disease.

## Introduction

Lung adenocarcinoma (LUAD) is a common histological subtype of lung cancer and remains a leading cause of cancer-related mortality worldwide [1,2]. Despite advances in early diagnosis and the development of targeted and immune-based therapies, the prognosis of patients with LUAD remains unsatisfactory, largely due to tumor heterogeneity, therapeutic resistance, and disease progression [3–6]. A deeper understanding of the molecular mechanisms underlying LUAD initiation and progression is therefore essential for identifying novel regulatory pathways and potential therapeutic targets.

Long noncoding RNAs (lncRNAs) are a class of RNA transcripts longer than 200 nucleotides that lack protein-coding capacity but exert regulatory functions at multiple levels of gene expression [7]. Accumulating evidence indicates that lncRNAs participate in diverse biological processes, including chromatin remodeling, transcriptional regulation, post-transcriptional control, and signal transduction [8–12]. In cancer, aberrant lncRNA expression has been associated with tumor cell proliferation, migration, invasion, and survival [13–16]. In LUAD, several lncRNAs have been reported to influence tumor-associated pathways; however, a large proportion of lncRNAs remain functionally uncharacterized, and their regulatory relevance in LUAD requires further investigation [17,18]. Recent studies have further highlighted the complex interplay between ncRNAs and epigenetic regulatory networks, suggesting that ncRNA-mediated signaling may contribute substantially to cancer heterogeneity and disease progression [19–21].

One widely recognized mechanism through which lncRNAs exert post-transcriptional regulation is by acting as competing endogenous RNAs (ceRNAs). Through sequence complementarity, lncRNAs can interact with microRNAs (miRNAs), thereby modulating the availability of miRNAs to bind their downstream targets [22–24]. This mode of regulation enables lncRNAs to indirectly influence gene expression networks without necessarily altering miRNA transcription [25]. Although numerous lncRNA–miRNA interactions have been described, the functional specificity and biological consequences of many such interactions in LUAD remain incompletely understood [26]. The competing endogenous RNA (ceRNA) model has become one of the most widely investigated mechanisms underlying lncRNA function in cancer biology. Increasing evidence suggests that dysregulated ceRNA networks contribute to the development and progression of lung cancer and may provide potential biomarkers or therapeutic targets for precision oncology [27].

RP3-340N1.2 is a lncRNA that has not been extensively studied in the context of lung cancer. Public transcriptomic datasets suggest that RP3-340N1.2 may be differentially expressed in tumor tissues; however, its expression pattern, biological relevance, and potential regulatory mechanisms in LUAD have not been systematically examined. Whether RP3-340N1.2 participates in miRNA-mediated regulatory networks and how such interactions might relate to LUAD-associated cellular behaviors remain open questions. Currently, RP3-340N1.2 remains largely uncharacterized, and no studies specifically investigating its biological functions in cancer or other pathological contexts were identified in publicly available literature databases at the time of manuscript revision. Therefore, its potential regulatory role in LUAD remains unclear.

In this study, we first analyzed the expression profile of RP3-340N1.2 in LUAD using publicly available datasets and validated its expression in LUAD cell lines. We then explored the functional consequences of RP3-340N1.2 modulation in vitro. Based on bioinformatic prediction using the LncBase Experimental v3 database, hsa-miR-4650-5p was identified as a candidate miRNA potentially interacting with RP3-340N1.2, and this interaction was further examined using molecular assays. In addition, downstream molecules associated with this regulatory axis were analyzed to provide insight into potential signaling pathways involved. Together, this work aims to characterize the expression and functional relevance of RP3-340N1.2 in LUAD and to preliminarily delineate a lncRNA–miRNA–associated regulatory framework.

## Materials and methods

### Data acquisition and public databases

RNA sequencing data and corresponding clinical information for lung adenocarcinoma (LUAD) were obtained from The Cancer Genome Atlas (TCGA) database through the Genomic Data Commons (GDC) portal (https://portal.gdc.cancer.gov/). Gene expression profiles were downloaded in fragments per kilobase per million mapped reads (FPKM) format and transformed into transcripts per million (TPM) values for downstream analyses. Log2(TPM + 1) transformation was applied where appropriate. The expression distribution of RP3-340N1.2 in LUAD and normal lung tissues was further validated using the GEPIA2 database (http://gepia2.cancer-pku.cn/), which integrates TCGA and GTEx datasets.

### Prediction of lncRNA–miRNA–mRNA regulatory network

Candidate microRNAs potentially interacting with RP3-340N1.2 were predicted using the LncBase Experimental v3 database (https://diana.e-ce.uth.gr/lncbasev3/home), which contains experimentally supported lncRNA–miRNA interactions.Putative downstream target genes of hsa-miR-4650-5p were predicted using three independent databases: miRDB, miRWalk, and TargetScan. Overlapping target genes among the three databases were selected for further analysis.Protein–protein interaction (PPI) networks were constructed using the STRING database (version 11.5), and functional enrichment analyses were performed based on the identified interacting proteins.

### Cell culture

Human lung adenocarcinoma cell lines A549, H1975, PC9, and H1299, as well as the normal human bronchial epithelial cell line BEAS-2B, were obtained from Procell Biotechnology Company (Wuhan, China). Cells were cultured in Dulbecco’s Modified Eagle Medium (DMEM; Gibco) or RPMI-1640 medium (Gibco) according to ATCC recommendations. All media were supplemented with 10% fetal bovine serum (FBS; Gibco, Cat. No. 10099−141) and 1% penicillin–streptomycin (Gibco). Cells were maintained at 37 °C in a humidified incubator containing 5% CO₂.

### RNA extraction and quantitative real-time PCR

Total RNA was extracted using TRIzol reagent (Invitrogen) according to the manufacturer’s instructions. RNA concentration and purity were measured using a NanoDrop spectrophotometer (Thermo Fisher Scientific). For lncRNA and mRNA analysis, complementary DNA (cDNA) was synthesized using the PrimeScript RT Reagent Kit (Takara). Quantitative PCR was performed using SYBR Premix Ex Taq II (Takara) on a QuantStudio Real-Time PCR System (Applied Biosystems). For miRNA detection, reverse transcription was performed using the Mir-X miRNA First-Strand Synthesis Kit (Takara), followed by quantitative PCR using TB Green Advantage qPCR Premix (Takara). GAPDH served as the internal control for lncRNA and mRNA, while U6 small nuclear RNA was used as the internal control for miRNA. Relative expression levels were calculated using the 2 ⁻ ΔΔCt method.

Primer sequences:

- RP3-340N1.2 F: CTTCTGGGCATCAATCACAG
- RP3-340N1.2 R: CATCCCTCTTCTATCACCTG
- SHC1 F: GAACAAGCTGAGTGGAGGCG
- SHC1 R: CCATGTACCGAACCAAGTAGGAA
- GAPDH F: GTCTCCTCTGACTTCAACAGCG
- GAPDH R: ACCACCCTGTTGCTGTAGCCAA
- miR-4650-5p Forward: TCAGGCCTCTTTCTACCTT
- U6 Forward: U6 5′ Primer (provided by kit)

### Cell transfection

Small interfering RNAs targeting RP3-340N1.2 (si-RP3-340N1.2), negative control siRNA (si-NC), hsa-miR-4650-5p mimics, miRNA negative control mimics, and miR-4650-5p inhibitors were purchased from GenePharma (Shanghai, China). The coding sequence of human SHC1 (NM_183001) was cloned into the pcDNA3.1 expression vector. The full-length RP3-340N1.2 sequence (ENSG00000227066) was amplified from human cDNA and inserted into the pcDNA3.1(+) vector. Cells were transfected using Lipofectamine 3000 (Invitrogen) according to the manufacturer’s protocol. Transfection efficiency was assessed by qRT-PCR 24–48 h after transfection.

Oligonucleotide sequences:

**Table:** 

Name	Sequence (5′ → 3′)
miR-4650-5p	UGAGUUGUAGUGGUUGUAUU
si-RP3-340N1.2-1	GCUCUAUGUUGGAAUACAUTT
si-RP3-340N1.2-2	CCAGAAUGGUGAAUCUAAATT
si-NC	Provided by manufacturer

### Cell proliferation, migration, and colony formation assays

Cell proliferation was assessed using the Cell Counting Kit-8 (CCK-8; Dojindo). Absorbance at 450 nm was measured using a microplate reader (Bio-Rad).Cell migration was evaluated using Transwell chambers with an 8-μm pore size (Corning). Wound-healing assays were performed by creating a linear scratch using sterile pipette tips, and images were captured at indicated time points.

For colony formation assays, cells were seeded into six-well plates and cultured for 10–14 days. Colonies were fixed with 4% paraformaldehyde (Solarbio) and stained with 0.1% crystal violet (Solarbio,).

### Dual-luciferase reporter assay

Wild-type and mutant fragments of RP3-340N1.2 or the SHC1 3′-UTR were cloned into the pmirGLO Dual-Luciferase miRNA Target Expression Vector (Promega). Cells were co-transfected with reporter plasmids and miRNA mimics using Lipofectamine 3000. Luciferase activity was measured 48 h after transfection using the Dual-Luciferase Reporter Assay System (Promega), according to the manufacturer’s instructions.

### RNA immunoprecipitation assay

RNA immunoprecipitation (RIP) assays were performed using the Magna RIP RNA-Binding Protein Immunoprecipitation Kit (Millipore). Anti-Ago2 antibody (Millipore) and normal mouse IgG (Millipore) were used for immunoprecipitation. Co-precipitated RNA was extracted and analyzed by qRT-PCR.

### Xenograft tumor model

All animal procedures were approved by the Animal Welfare and Ethics Review Committee of the Affiliated Hospital of Guizhou Medical University (approval number: 2402333) and were performed in accordance with institutional guidelines for laboratory animal care and use. Female BALB/c nude mice aged 4–6 weeks were obtained from an accredited animal facility and housed under specific pathogen-free conditions at 22 ± 2°C with a 12 h light/dark cycle and free access to food and water. H1975 cells transfected with si-NC or si-RP3-340N1.2 were collected during the logarithmic growth phase, washed with sterile PBS, and resuspended in serum-free medium. A total of 5 × 10⁶ cells in 100 μL serum-free medium were subcutaneously injected into the flank of each mouse to establish xenograft tumors. For cell implantation, mice were anesthetized with 2% isoflurane inhalation in oxygen. Mice were randomly divided into the si-NC and si-RP3-340N1.2 groups, with three mice in each group. Tumor size and body weight were measured every 3–4 days. Tumor volume was calculated using the formula: V = (length × width²)/2. At approximately 28 days after injection, mice were euthanized, and tumor tissues were excised, photographed, weighed, and collected for subsequent analysis.

### Humane endpoints and animal welfare

Humane endpoints were predefined to minimize animal suffering. Mice were monitored daily for general appearance, activity, posture, grooming behavior, food intake, body weight, and tumor burden. Animals were euthanized before the planned endpoint if any of the following criteria were met: tumor diameter exceeding 1.5 cm, body weight loss greater than 20%, impaired mobility, persistent lethargy, ulceration or necrosis at the tumor site, severe distress, or any other condition judged by trained personnel to compromise animal welfare. No unexpected deaths occurred during the experimental period. At the experimental endpoint or when humane endpoint criteria were met, mice were euthanized by carbon dioxide inhalation using a gradual-fill method at a displacement rate of 20–30% of the chamber volume per minute. After cessation of respiration, cervical dislocation was performed as a secondary physical method to ensure death. All animal procedures were performed by trained personnel in accordance with approved institutional animal welfare regulations.

### Western blot analysis

Total protein was extracted using RIPA lysis buffer (Beyotime, Cat. No. P0013B) supplemented with protease and phosphatase inhibitor cocktail (Beyotime, Cat. No. P1045). Protein concentration was determined using the BCA Protein Assay Kit (Thermo Fisher Scientific, Cat. No. 23225).

Proteins were separated by SDS–PAGE and transferred onto PVDF membranes (Millipore, Cat. No. IPVH00010). Membranes were incubated with primary antibodies against SHC1 (Proteintech, Cat. No. 67194–22-lg), phospho-ERK1/2 (Thr202/Tyr204) (Proteintech, Cat. No. 28733–1-AP), ERK1/2 (Proteintech, Cat. No. 66192–1-lg), and β-actin (Proteintech, Cat. No. 20536–1-lg). HRP-conjugated secondary antibodies were obtained from Cell Signaling Technology. Signals were visualized using enhanced chemiluminescence (ECL) reagent.

Western blot band intensities were quantified using ImageJ software. For total protein analysis, target protein gray values were normalized to β-actin from the same sample, and the control group was normalized to 1. For ERK1/2 phosphorylation analysis, relative p-ERK1/2/ERK1/2 levels were calculated as (p-ERK1/2/β-actin)/(ERK1/2/β-actin), followed by normalization of the control group to 1. Quantification was performed using data obtained from at least three independent biological replicates. Original uncropped western blot images are available in S1 Fig.

### Statistical analysis

All statistical analyses were performed using R software (version 4.0.5) and GraphPad Prism 9.0 (GraphPad Software, USA). Comparisons between two groups were conducted using Student’s t-test, while multiple group comparisons were analyzed using one-way ANOVA. Correlation analyses were performed using Spearman’s rank correlation coefficient. All in vitro experiments were independently repeated at least three times. Data are presented as mean ± standard deviation (SD). A p-value < 0.05 was considered statistically significant. The numerical data underlying all quantitative analyses are provided in S1 Data.

## Results

### RP3-340N1.2 is upregulated in LUAD and lung cancer cell lines

**Fig 1:**
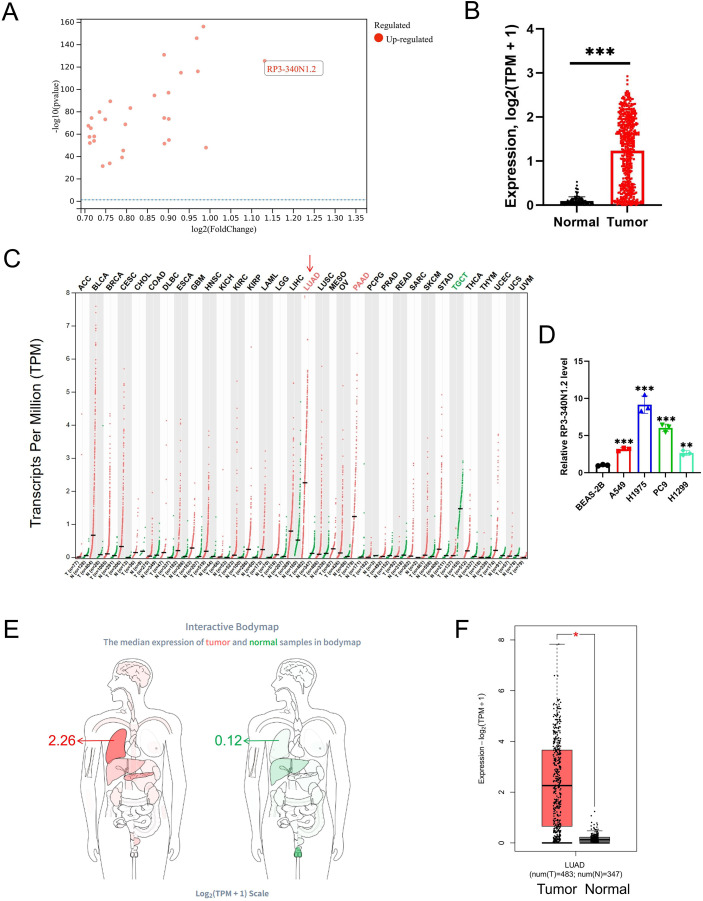
Expression profiling of RP3-340N1.2 in lung adenocarcinoma. (A) Volcano plot of differentially expressed long non-coding RNAs generated from RNA-sequencing data of the TCGA-LUAD cohort, comparing tumor tissues with normal lung tissues. RP3-340N1.2 is indicated. (B) Distribution of RP3-340N1.2 expression levels in tumor and normal lung tissues from the TCGA-LUAD cohort (tumor, n = 483; normal, n = 347). Expression values are shown as log2(TPM + 1). (C) Pan-cancer expression profile of RP3-340N1.2 across multiple cancer types based on TCGA datasets. Each dot represents an individual sample, and expression values are shown as transcripts per million (TPM). (D) Quantitative real-time PCR analysis of RP3-340N1.2 expression in lung cancer cell lines (A549, H1975, PC9, and H1299) and the normal human bronchial epithelial cell line BEAS-2B. Data represent mean ± SD from three independent experiments. (E) Tissue distribution of RP3-340N1.2 expression in lung adenocarcinoma samples retrieved from the GEPIA2 database (http://gepia2.cancer-pku.cn/) based on TCGA-LUAD data. (F) Expression distribution of RP3-340N1.2 in lung adenocarcinoma analyzed using an integrated dataset combining TCGA-LUAD and GTEx-LUAD cohorts, obtained from the GEPIA2 database. Statistical significance was calculated using the methods described in the Materials and Methods section. *P < 0.05, **P < 0.01, ***P < 0.001.

To identify dysregulated long non-coding RNAs in lung adenocarcinoma (LUAD), we first analyzed RNA-sequencing data from The Cancer Genome Atlas Lung Adenocarcinoma (TCGA-LUAD) cohort, which includes 483 tumor samples and 347 normal lung tissues. Differential expression analysis revealed a subset of lncRNAs that were significantly upregulated in LUAD, among which RP3-340N1.2 was highlighted due to its relatively high fold change and statistical significance (Fig 1A). Consistent with these findings, RP3-340N1.2 expression was markedly elevated in LUAD tumor tissues compared with normal lung tissues in the TCGA-LUAD cohort when visualized as log2(TPM + 1) values (Fig 1B), indicating aberrant upregulation of RP3-340N1.2 at the transcriptomic level. To further characterize the tissue distribution of RP3-340N1.2 in LUAD, expression data were retrieved from the GEPIA2 database (http://gepia2.cancer-pku.cn/), which integrates TCGA RNA-seq data. Analysis of TCGA-LUAD samples demonstrated that RP3-340N1.2 expression was markedly higher in lung adenocarcinoma tissues compared with normal lung tissues, confirming its tumor-enriched expression pattern (Fig 1E). In addition, we examined RP3-340N1.2 expression using a larger integrated dataset through GEPIA2, which combines TCGA-LUAD and GTEx-LUAD cohorts. Consistently, RP3-340N1.2 expression remained significantly elevated in LUAD tumor samples relative to normal controls in the merged analysis (Fig 1F), further supporting the robustness of its upregulation across independent datasets.

We next evaluated RP3-340N1.2 expression in vitro using a panel of human lung cancer cell lines. Quantitative real-time PCR analysis showed that RP3-340N1.2 expression was significantly higher in A549, H1975, PC9, and H1299 lung cancer cells compared with the normal human bronchial epithelial cell line BEAS-2B (Fig 1D). Notably, RP3-340N1.2 expression levels varied among different LUAD cell lines, suggesting potential intratumoral heterogeneity. Collectively, analyses based on the TCGA-LUAD cohort, GEPIA2-integrated datasets, and lung cancer cell models consistently demonstrate that RP3-340N1.2 is upregulated in LUAD, providing a strong rationale for further investigation of its functional role in lung adenocarcinoma. Among the examined LUAD cell lines, H1975 and PC9 cells exhibited relatively higher endogenous RP3-340N1.2 expression levels compared with A549 and H1299 cells. Therefore, these two cell lines were selected for subsequent loss-of-function and mechanistic experiments to facilitate evaluation of RP3-340N1.2-associated biological effects.

### Knockdown of RP3-340N1.2 suppresses proliferation, migration, and tumor growth in lung adenocarcinoma cells

**Fig 2:**
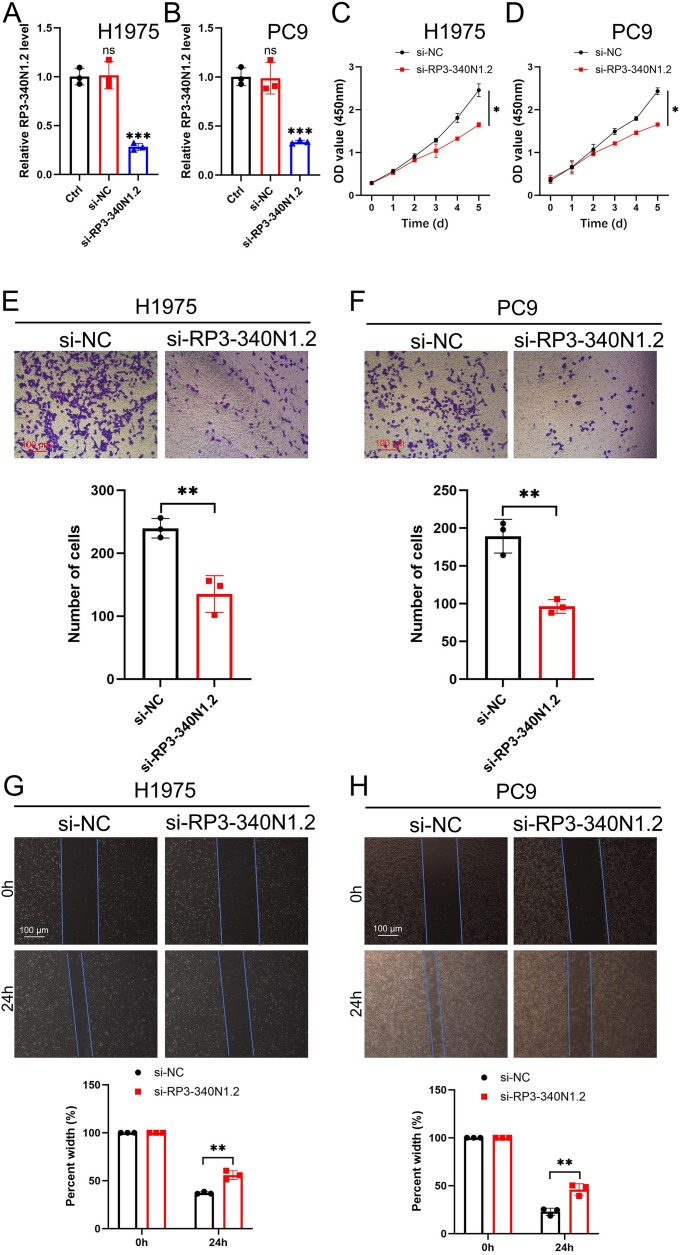
Effects of RP3-340N1.2 knockdown on proliferation and migration-associated behaviors in lung adenocarcinoma cells. (A, B) Relative expression levels of RP3-340N1.2 in H1975 and PC9 cells after transfection with si-NC or si-RP3-340N1.2, as determined by quantitative real-time PCR. (C, D) Cell proliferation of H1975 and PC9 cells measured by CCK-8 assays at the indicated time points. (E, F) Representative images and quantification of Transwell migration assays in H1975 and PC9 cells. Scale bar, 100 μm. (G, H) Wound healing assays in H1975 and PC9 cells at 0 h and 24 h after scratching. Wound width was quantified as the percentage of the initial gap width. Scale bar, 100 μm. Data are presented as mean ± SD. In vitro experiments were performed using three independent biological replicates. Statistical analysis was performed using Student’s t-test or one-way ANOVA as appropriate. ns, not significant; *P < 0.05, **P < 0.01, ***P < 0.001.

To further evaluate the biological relevance of RP3-340N1.2 in lung adenocarcinoma, small interfering RNAs targeting RP3-340N1.2 were transfected into H1975 and PC9 cells. Quantitative real-time PCR analysis confirmed that RP3-340N1.2 expression was effectively reduced after si-RP3-340N1.2 transfection, whereas no obvious difference was observed between the Ctrl and si-NC groups (Fig 2A,B). CCK-8 assays demonstrated that suppression of RP3-340N1.2 was associated with a reduced proliferative tendency in both H1975 and PC9 cells across the indicated time points (Fig 2C,D). The influence of RP3-340N1.2 on cell motility was further assessed. Transwell migration assays demonstrated that the number of migrated cells was lower in the si-RP3-340N1.2 group than in the si-NC group in both H1975 and PC9 cells (Fig 2E,F). Similarly, wound healing assays showed a slower wound closure rate at 24 h following RP3-340N1.2 silencing (Fig 2G,H), indicating altered migration-associated behavior.

**Fig 3:**
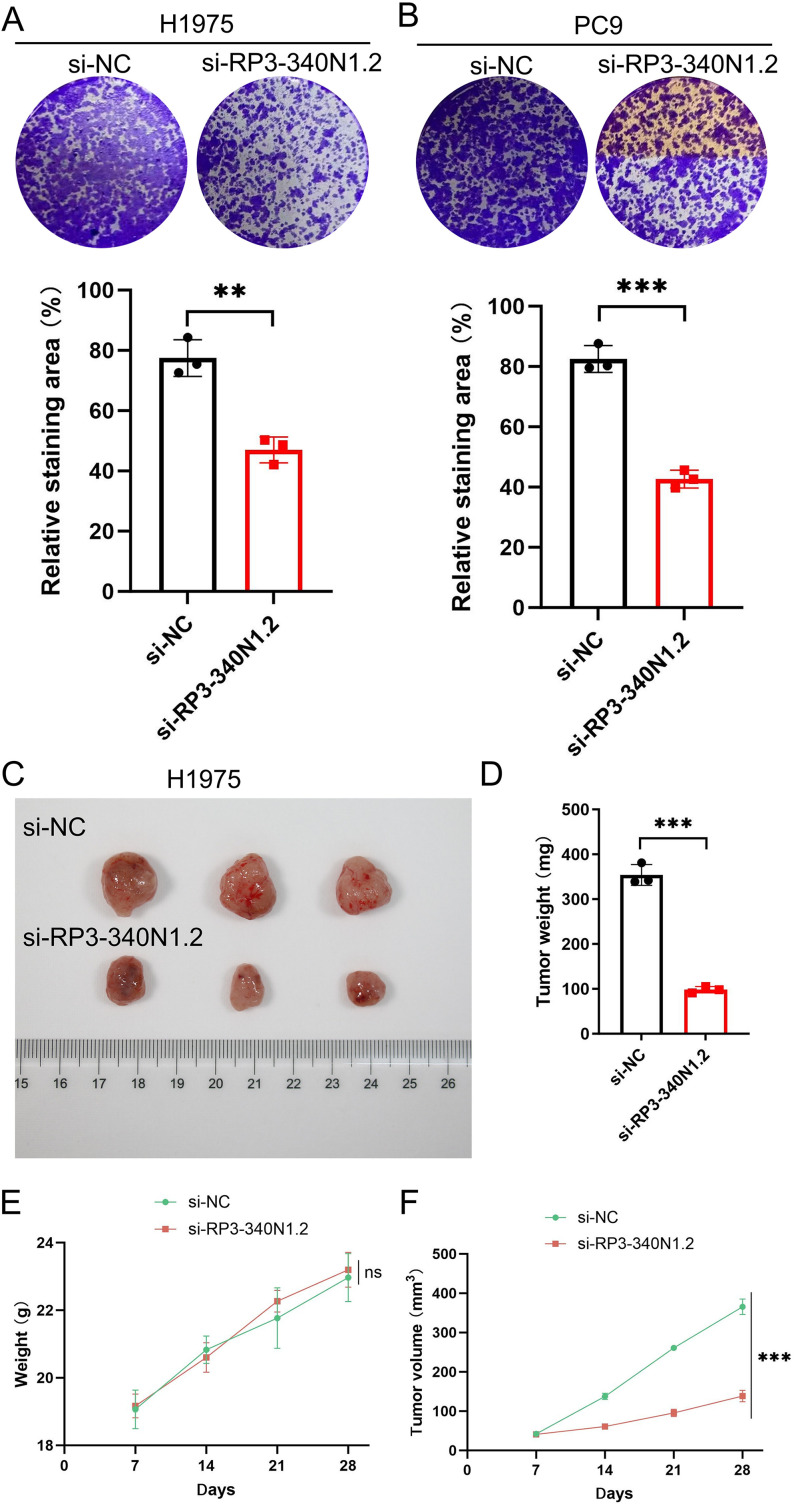
Effects of RP3-340N1.2 knockdown on clonogenic growth and xenograft tumor formation. (A, B) Colony formation assays and quantitative analysis of clonogenic growth in H1975 and PC9 cells. (C) Representative images of xenograft tumors generated from H1975 cells transfected with si-NC or si-RP3-340N1.2. (D) Endpoint tumor weight measurements of nude mice bearing H1975-derived xenografts. (E) Body weight curves of nude mice during the experimental period. (F) Tumor volume curves of xenograft tumors at the indicated time points. Tumor volume was calculated using the formula: volume = length × width²/ 2. Data are presented as mean ± SD. Xenograft experiments were performed with three mice per group. Statistical analysis was performed using Student’s t-test or one-way ANOVA as appropriate. ns, not significant; **P < 0.01, ***P < 0.001.

To further investigate the effect of RP3-340N1.2 on long-term proliferative capacity, colony formation assays were performed in H1975 and PC9 cells. RP3-340N1.2 knockdown was associated with reduced clonogenic growth compared with the si-NC group in both cell lines (Fig 3A,B). To further extend these findings in vivo, a xenograft tumor model was established using H1975 cells transfected with si-NC or si-RP3-340N1.2. Representative tumor images showed that tumors derived from RP3-340N1.2-silenced cells were visibly smaller than those in the control group (Fig 3C). Tumor growth curve analysis further demonstrated a slower increase in tumor volume in the si-RP3-340N1.2 group during the observation period, whereas body weight did not show an obvious difference between groups (Fig 3E,F). In addition, tumor weights at the experimental endpoint were lower in the si-RP3-340N1.2 group compared with the control group (Fig 3D). Collectively, these findings suggest that reduced RP3-340N1.2 expression is associated with attenuated proliferative and migration-related phenotypes in vitro and decreased tumor growth in vivo.

### RP3-340N1.2 interacts with miR-4650-5p in lung adenocarcinoma cells

**Fig 4:**
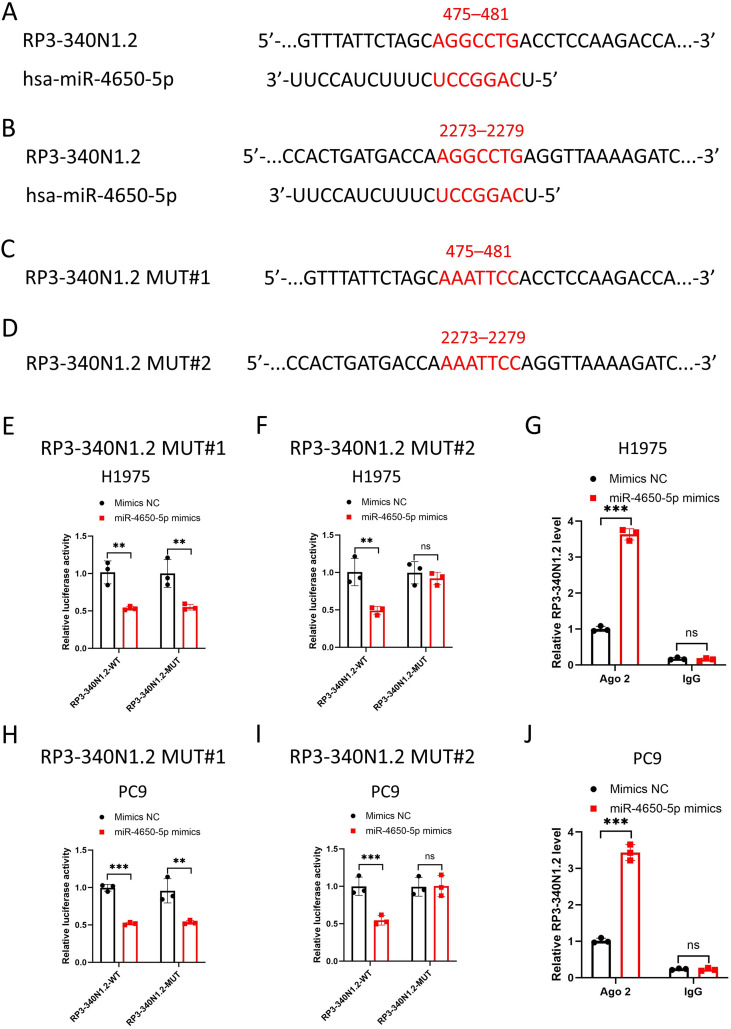
Identification and validation of the interaction between RP3-340N1.2 and miR-4650-5p. (A, B) Candidate miRNAs potentially associated with RP3-340N1.2 and predicted interaction regions were obtained from the LncBase Experimental v3 database (https://diana.e-ce.uth.gr/lncbasev3/home). The positions of the putative miR-4650-5p–complementary regions within RP3-340N1.2 are indicated. (C, D) Schematic representation of mutant RP3-340N1.2 reporter constructs (MUT#1 and MUT#2) with substitutions introduced into the indicated regions. (E, F) Dual-luciferase reporter assays in H1975 cells co-transfected with RP3-340N1.2 wild-type or mutant reporters and miR-4650-5p mimics or negative control mimics. (G) Ago2 RNA immunoprecipitation (RIP) in H1975 cells followed by qPCR detection of RP3-340N1.2 in Ago2 and IgG immunoprecipitates. (H, I) Dual-luciferase reporter assays in PC9 cells co-transfected with RP3-340N1.2 wild-type or mutant reporters and miR-4650-5p mimics or negative control mimics. (J) Ago2 RIP in PC9 cells followed by qPCR detection of RP3-340N1.2 in Ago2 and IgG immunoprecipitates. Data are presented as mean ± SD from three independent experiments. Statistical analyses were performed as described in the Materials and Methods section. ns, not significant, *P < 0.05, **P < 0.01, ***P < 0.001.

To identify candidate microRNAs that may interact with RP3-340N1.2, we first queried the LncBase Experimental v3 database and focused on miRNAs with potential experimentally supported associations. Based on this analysis, hsa-miR-4650-5p was selected for subsequent validation, and two putative complementary regions within the RP3-340N1.2 sequence were delineated (nucleotides 475–481 and 2273–2279) (Fig 4A-B). To test whether these predicted regions were required for the interaction, we constructed reporter plasmids containing the wild-type RP3-340N1.2 fragments as well as corresponding mutants in which each predicted binding region was altered (MUT#1 for site 475–481; MUT#2 for site 2273–2279) (Fig 4C-D). Dual-luciferase reporter assays were then performed in H1975 cells co-transfected with miR-4650-5p mimics or negative control mimics. The reporter assays were used to evaluate whether miR-4650-5p could affect luciferase activity in a manner dependent on the predicted RP3-340N1.2 binding regions (Fig 4E-F). The same reporter strategy was further assessed in PC9 cells to examine the reproducibility of the interaction pattern across LUAD cell contexts (Fig 4H-I). Finally, to explore whether RP3-340N1.2 could be detected in the RNA-induced silencing complex in the presence of miR-4650-5p, an Ago2-RIP assay was conducted in both H1975 and PC9 cells followed by qPCR detection of RP3-340N1.2 in Ago2 versus IgG immunoprecipitates (Fig 4G-J). Collectively, these experiments provide evidence supporting an interaction between RP3-340N1.2 and miR-4650-5p in lung adenocarcinoma cells, and identify a candidate interaction site for downstream mechanistic analyses.

### SHC1 is identified as a candidate downstream target of miR-4650-5p and miR-4650-5p modulates LUAD cell proliferation and migration

**Fig 5:**
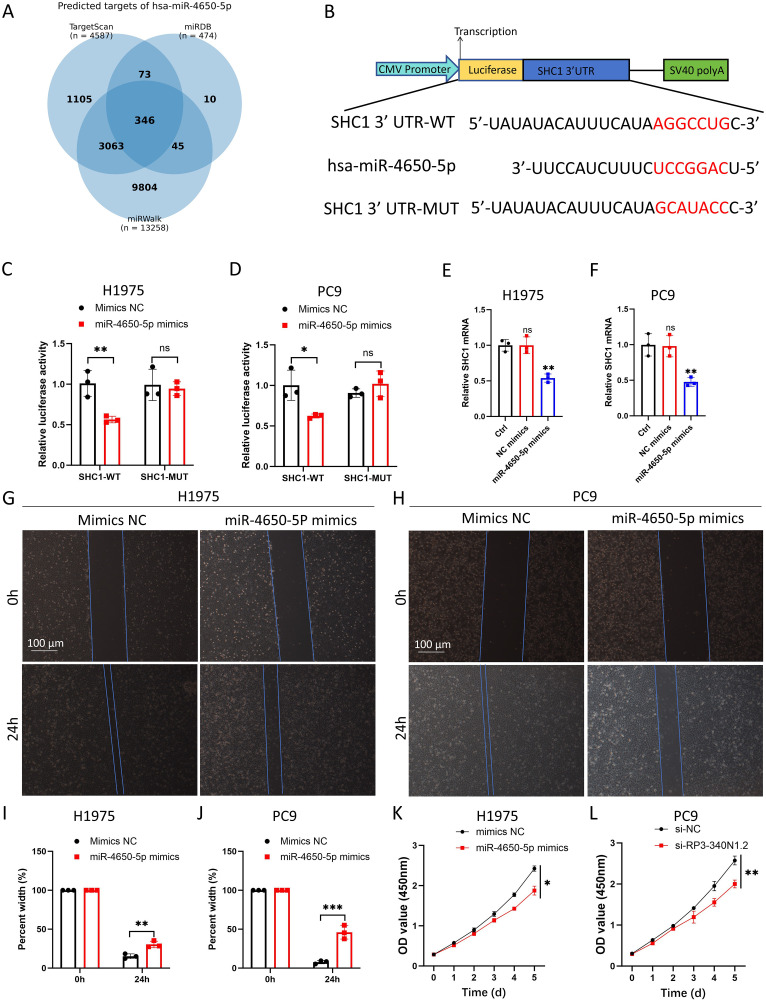
Identification of SHC1 as a candidate downstream target of miR-4650-5p and functional assessment of miR-4650-5p in LUAD cells. (A) Venn diagram showing the overlap of predicted miR-4650-5p target genes obtained from the miRDB, miRWalk, and TargetScan databases. (B) Schematic illustration of the luciferase reporter constructs containing the wild-type (WT) or mutant (MUT) SHC1 3′UTR with the predicted miR-4650-5p binding sequence indicated. (C, D) Dual-luciferase reporter assays performed in H1975 (C) and PC9 (D) cells co-transfected with SHC1 3′UTR-WT or SHC1 3′UTR-MUT reporters and miR-4650-5p mimics or negative control mimics. (E, F) Quantitative real-time PCR analysis of SHC1 mRNA expression in H1975 (E) and PC9 (F) cells following transfection with miR-4650-5p mimics or negative control mimics. (G, H) Representative images of wound-healing assays in H1975 (G) and PC9 (H) cells following transfection with miR-4650-5p mimics or negative control mimics. Images were captured at 0 h and 24 h. Scale bar, 100 μm. (I, J) Quantitative analysis of wound width in H1975 (I) and PC9 (J) cells based on the wound-healing assays. (K, L) CCK-8 assays showing cell proliferation in H1975 (K) and PC9 (L) cells following transfection with miR-4650-5p mimics or negative control mimics at the indicated time points. Data are presented as mean ± SD from three independent biological replicates. Statistical analyses were performed using Student’s t-test or one-way ANOVA as appropriate. ns, not significant, *P < 0.05, **P < 0.01, ***P < 0.001.

To identify candidate downstream target genes of miR-4650-5p, potential mRNA targets were first predicted using three independent databases, including miRDB, miRWalk, and TargetScan. The intersection of the predicted target sets yielded 26 overlapping candidate genes for subsequent analysis (Fig 5A). The relevant information about Venn diagrams is presented in S1 Table. Among these candidates, SHC1 was selected for further validation based on its predicted miR-4650-5p binding site within the 3′ untranslated region (3′UTR) and its known relevance to receptor tyrosine kinase-related signaling. A schematic representation of the wild-type SHC1 3′UTR fragment containing the predicted miR-4650-5p complementary sequence, together with the corresponding mutant construct, is shown (Fig 5B). Dual-luciferase reporter assays were then performed to assess whether miR-4650-5p could interact with the SHC1 3′UTR. In H1975 and PC9 cells, transfection with miR-4650-5p mimics reduced the luciferase activity of the SHC1 3′UTR-WT reporter, whereas this effect was not observed in cells transfected with the mutant reporter (Fig 5C,D). In parallel, quantitative real-time PCR analysis showed that miR-4650-5p overexpression was associated with reduced endogenous SHC1 mRNA expression in both H1975 and PC9 cells (Fig 5E,F). These results support SHC1 as a candidate downstream target associated with miR-4650-5p in LUAD cells.

To further address the functional relevance of miR-4650-5p itself, additional gain-of-function experiments were performed using miR-4650-5p mimics. Wound-healing assays showed that miR-4650-5p overexpression slowed wound closure in both H1975 and PC9 cells compared with the mimics NC group (Fig 5G–J). CCK-8 assays further demonstrated that miR-4650-5p overexpression was associated with reduced cell proliferation over the indicated time points in both cell lines (Fig 5K,L). Collectively, these findings indicate that miR-4650-5p participates in the regulation of LUAD cell proliferation and migration and further supports its involvement in the RP3-340N1.2/miR-4650-5p/SHC1 regulatory framework.

### Bioinformatic characterization of SHC1-associated clinical outcomes and signaling pathways

**Fig 6:**
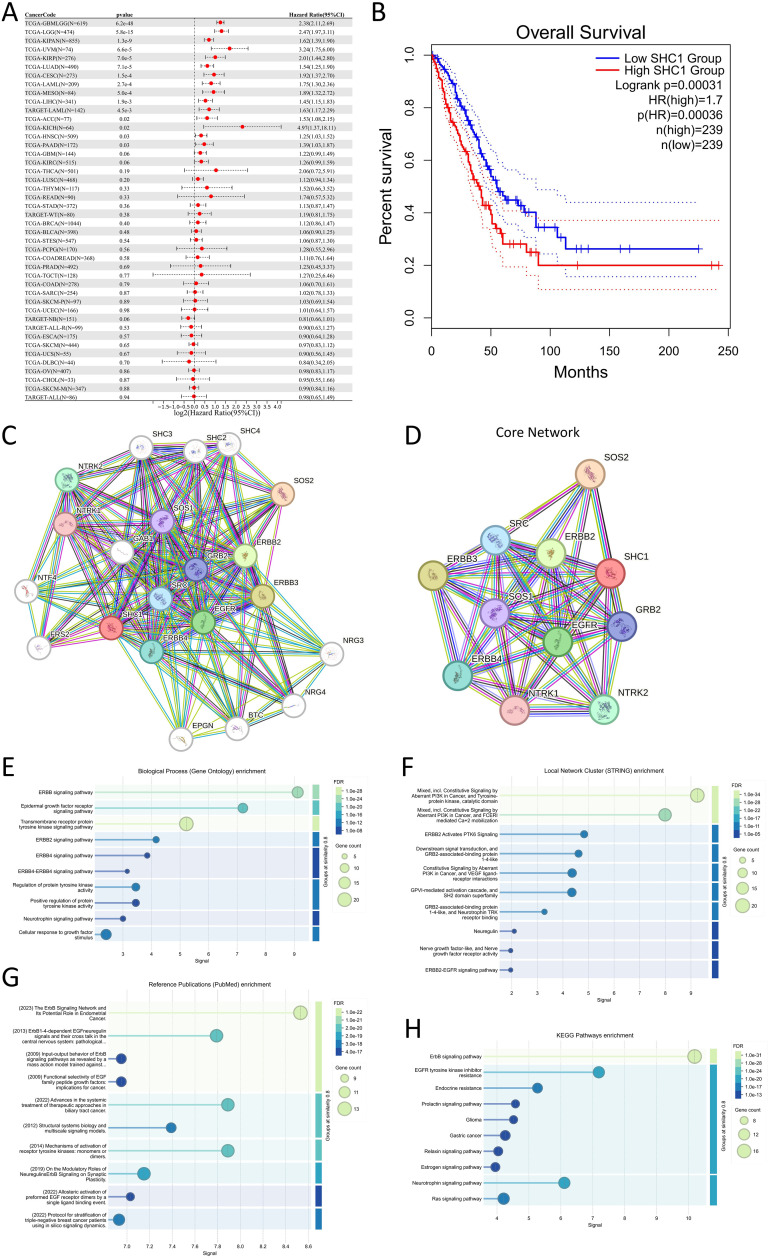
Clinical relevance and functional annotation of SHC1 based on public datasets. (A) Pan-cancer Cox regression analysis of SHC1 expression across multiple cancer types, showing hazard ratios with 95% confidence intervals. (B) Kaplan–Meier overall survival analysis of lung adenocarcinoma patients stratified by SHC1 expression level. (C) Protein–protein interaction (PPI) network of SHC1 constructed using the STRING database. D) Core interaction subnetwork centered on SHC1 extracted from the PPI network. E) Gene Ontology (GO) biological process enrichment analysis of SHC1-associated genes. (F) Local network cluster enrichment analysis of SHC1-associated proteins based on STRING annotations. (G) Literature-based enrichment analysis of SHC1-associated genes using PubMed reference annotations. (H) KEGG pathway enrichment analysis of SHC1-associated genes. All analyses were performed using publicly available datasets and bioinformatics tools. Statistical thresholds and analysis parameters are described in the Materials and Methods section.

To further explore the potential clinical relevance of SHC1 in human cancers, survival analyses were performed across multiple tumor types using publicly available datasets (TCGA-LUAD). A pan-cancer Cox regression analysis revealed heterogeneous associations between SHC1 expression and patient survival outcomes among different cancer types (Fig 6A). In lung adenocarcinoma, Kaplan–Meier survival analysis showed that patients stratified according to SHC1 expression levels exhibited distinct overall survival patterns, with SHC1 expression serving as a prognostic indicator in this cohort (Fig 6B). To investigate the functional context in which SHC1 may operate, protein–protein interaction (PPI) networks were constructed using the STRING database. SHC1 was found to be embedded within a densely connected interaction network involving multiple receptor tyrosine kinases and adaptor proteins (Fig 6C). A core subnetwork centered on SHC1 was subsequently extracted to highlight its immediate interacting partners (Fig 6D). Gene Ontology (GO) enrichment analysis of the SHC1-associated network indicated enrichment in biological processes related to receptor-mediated signaling and intracellular signal transduction (Fig 6E). Consistently, local network cluster enrichment analysis further suggested associations with pathways involved in signal relay downstream of growth factor receptors (Fig 6F). In addition, literature-based enrichment analysis identified multiple publications linking SHC1-related signaling modules to cancer-associated biological processes (Fig 6G). KEGG pathway enrichment analysis further revealed that SHC1-associated genes were enriched in pathways related to receptor tyrosine kinase signaling and downstream oncogenic signaling cascades (Fig 6H). Together, these analyses provide a contextual framework for understanding the potential involvement of SHC1 in lung adenocarcinoma–associated signaling networks and clinical outcomes.

### Association of RP3-340N1.2 with miR-4650-5p–related SHC1 expression and ERK signaling

**Fig 7:**
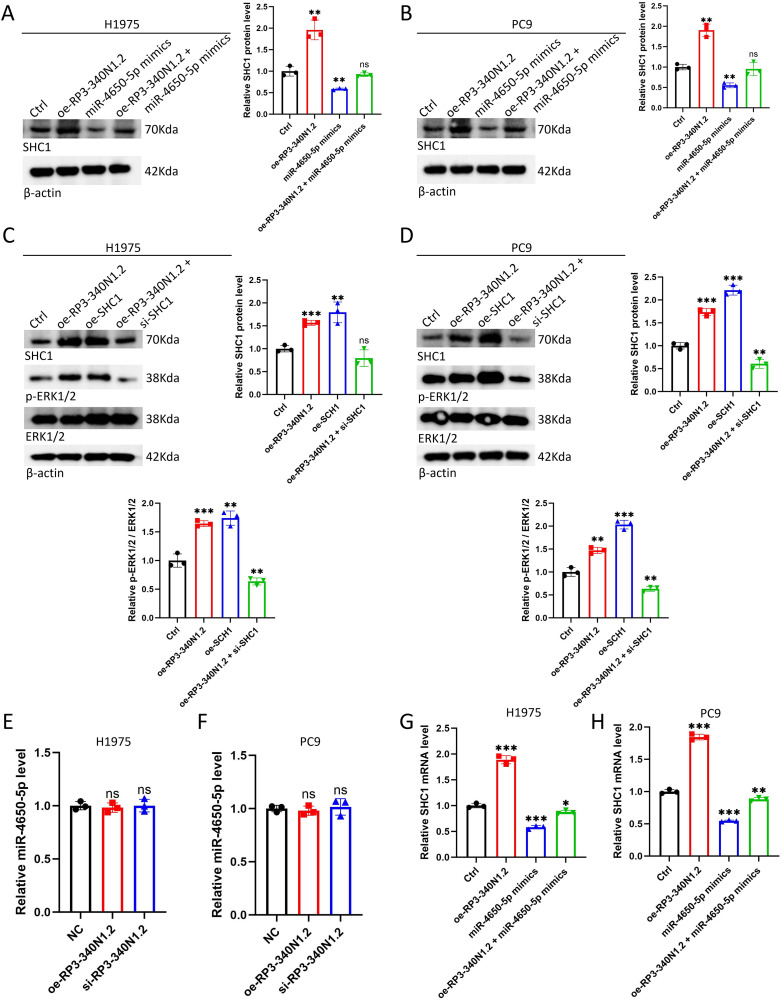
Association of RP3-340N1.2 with miR-4650-5p-related SHC1 expression and ERK1/2 phosphorylation. (A, B) Western blot analysis and densitometric quantification of SHC1 protein levels in H1975 (A) and PC9 (B) cells transfected with control vector, RP3-340N1.2 overexpression plasmid, miR-4650-5p mimics, or RP3-340N1.2 overexpression plasmid combined with miR-4650-5p mimics. β-actin was used as the loading control. (C, D) Western blot analysis and densitometric quantification of SHC1, phosphorylated ERK1/2 (p-ERK1/2), total ERK1/2, and β-actin in H1975 (C) and PC9 (D) cells transfected with control vector, RP3-340N1.2 overexpression plasmid, SHC1 overexpression plasmid, or RP3-340N1.2 overexpression plasmid combined with si-SHC1. (E, F) qRT-PCR analysis of endogenous miR-4650-5p expression in H1975 (E) and PC9 (F) cells after RP3-340N1.2 overexpression or knockdown. (G, H) qRT-PCR analysis of endogenous SHC1 mRNA expression in H1975 (G) and PC9 (H) cells transfected with control vector, RP3-340N1.2 overexpression plasmid, miR-4650-5p mimics, or RP3-340N1.2 overexpression plasmid combined with miR-4650-5p mimics. Western blot band intensities were quantified using ImageJ. For total protein analysis, target protein levels were normalized to β-actin and then normalized to the control group, which was set to 1. For ERK1/2 phosphorylation analysis, relative p-ERK1/2/ERK1/2 levels were calculated as (p-ERK1/2/β-actin)/(ERK1/2/β-actin), followed by normalization to the control group. Data are presented as mean ± SD from three independent biological replicates. Statistical analysis was performed using Student’s t-test or one-way ANOVA followed by appropriate post hoc tests. ns, not significant, *P < 0.05, **P < 0.01, ***P < 0.001.

To examine whether RP3-340N1.2 was associated with miR-4650-5p-related regulation of SHC1 expression, RP3-340N1.2 overexpression and miR-4650-5p mimic transfection were performed in H1975 and PC9 cells. Western blot analysis showed that RP3-340N1.2 overexpression increased SHC1 protein abundance, whereas miR-4650-5p mimics reduced SHC1 protein expression. Co-transfection with miR-4650-5p mimics largely counteracted the RP3-340N1.2-associated increase in SHC1 protein levels, resulting in SHC1 expression close to that of the control group (Fig 7A,B).

To further determine whether RP3-340N1.2 altered total miR-4650-5p abundance, qRT-PCR was performed after RP3-340N1.2 overexpression or knockdown. RP3-340N1.2 modulation did not significantly change endogenous miR-4650-5p levels in either H1975 or PC9 cells (Fig 7E,F). This finding is consistent with a ceRNA-related model in which RP3-340N1.2 may affect miR-4650-5p availability or activity rather than its total expression level.

We next examined SHC1 mRNA expression under the same four-group experimental design. RP3-340N1.2 overexpression increased SHC1 mRNA expression, while miR-4650-5p mimics decreased SHC1 mRNA levels. In the combined oe-RP3-340N1.2 + miR-4650-5p mimics group, the RP3-340N1.2-associated increase in SHC1 mRNA was largely reduced in both H1975 and PC9 cells (Fig 7G,H). These results further support a miR-4650-5p-related association between RP3-340N1.2 and endogenous SHC1 expression.

To explore whether SHC1 changes were accompanied by downstream signaling alterations, SHC1 overexpression or knockdown was introduced in the context of RP3-340N1.2 overexpression. In H1975 and PC9 cells, RP3-340N1.2 overexpression was associated with increased SHC1 protein levels and elevated ERK1/2 phosphorylation. SHC1 overexpression further enhanced SHC1 abundance and p-ERK1/2 levels, whereas SHC1 knockdown reduced both SHC1 expression and ERK1/2 phosphorylation under RP3-340N1.2-overexpressing conditions (Fig 7C,D). Total ERK1/2 levels remained relatively stable.

Collectively, these findings suggest that RP3-340N1.2 is associated with miR-4650-5p-related regulation of SHC1 expression and may be linked to downstream ERK1/2 phosphorylation in LUAD cells.

### SHC1 contributes to RP3-340N1.2-associated proliferative phenotypes

**Fig 8:**
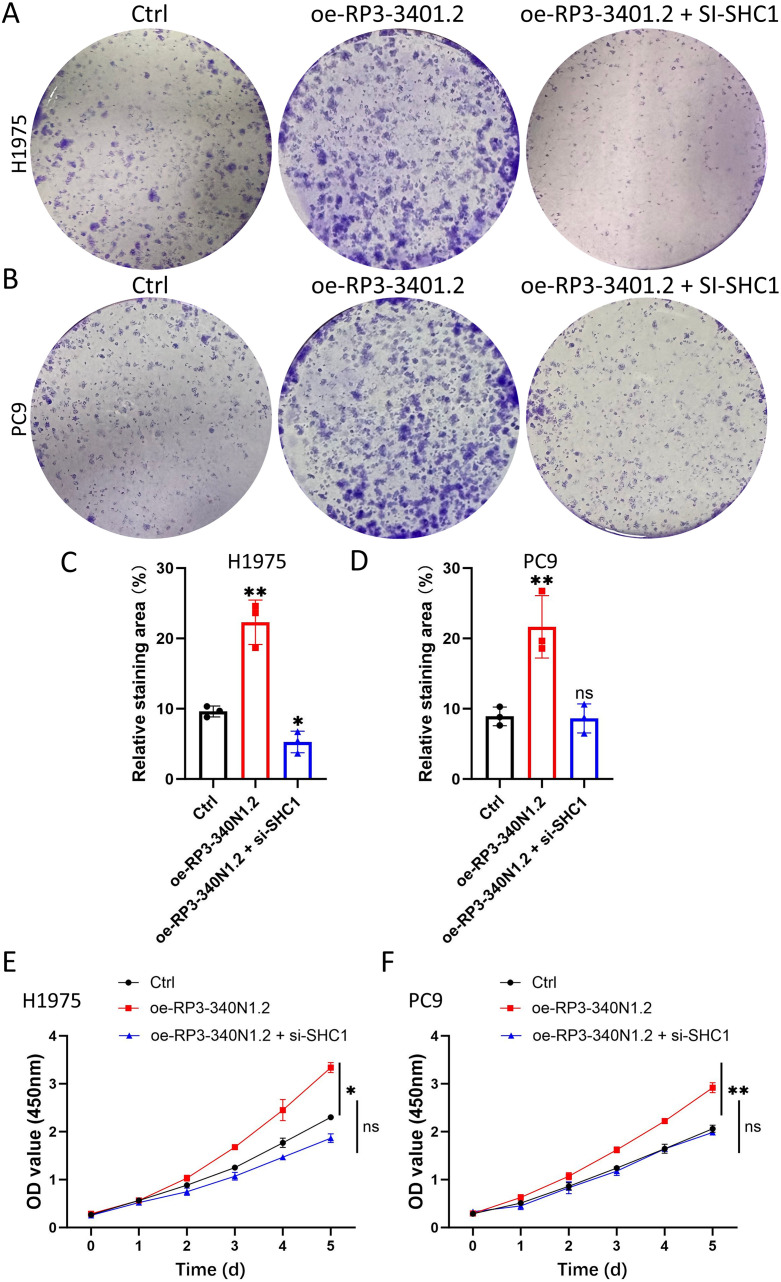
SHC1 knockdown counteracts RP3-340N1.2-associated proliferative phenotypes. (A-D) Representative images (A, B) and quantitative analysis (C, D) of colony formation assays performed in H1975 (A) and PC9 (B) cells transfected with control vector, RP3-340N1.2 overexpression plasmid, or RP3-340N1.2 overexpression plasmid combined with si-SHC1. (E, F) CCK-8 assays showing OD450 values at the indicated time points in H1975 (E) and PC9 (F) cells under the same transfection conditions. Data are presented as mean ± SD from three independent biological replicates. Statistical analyses were performed using one-way ANOVA followed by appropriate post hoc tests. ns, not significant, *P < 0.05, **P < 0.01.

To further evaluate whether SHC1 is involved in RP3-340N1.2-associated proliferative effects, dependency assays were performed in H1975 and PC9 cells. Colony formation assays showed that RP3-340N1.2 overexpression increased clonogenic growth compared with the control group, whereas simultaneous SHC1 knockdown markedly reduced this effect (Fig 8A-D). The clonogenic capacity in the oe-RP3-340N1.2 + si-SHC1 group was comparable to or lower than that in the control group, suggesting that SHC1 depletion can counteract the proliferative changes associated with RP3-340N1.2 overexpression.

Consistently, CCK-8 assays showed that RP3-340N1.2 overexpression was associated with increased cell growth over time in both H1975 and PC9 cells (Fig 8E,F). Co-transfection with si-SHC1 substantially reduced this growth-promoting tendency. The proliferation curves of the oe-RP3-340N1.2 + si-SHC1 group were close to those of the control group, further supporting the involvement of SHC1 in RP3-340N1.2-associated proliferative phenotypes.

Together, these findings suggest that SHC1 contributes to the proliferative effects associated with RP3-340N1.2 overexpression and may act as an important downstream component within this regulatory framework.

### SHC1 contributes to RP3-340N1.2-associated migratory and invasive phenotypes

**Fig 9:**
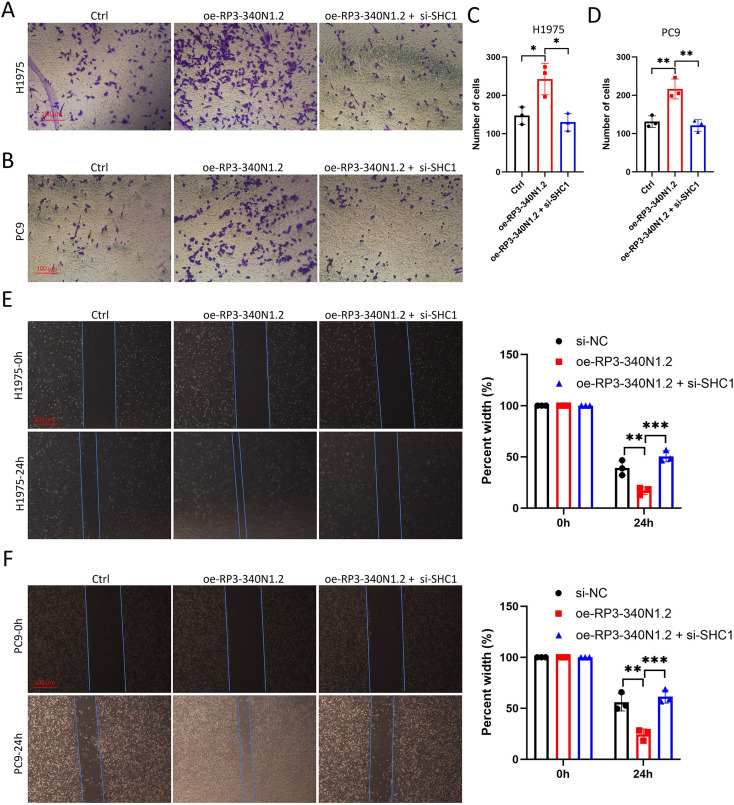
SHC1 knockdown counteracts RP3-340N1.2-associated migratory and invasion-related phenotypes. (A-D) Representative images (A, B) and quantitative analysis (C, D) of Transwell assays performed in H1975 (A) and PC9 (B) cells transfected with control vector (Ctrl), RP3-340N1.2 overexpression plasmid (oe-RP3-340N1.2), or oe-RP3-340N1.2 combined with si-SHC1. Scale bar, 100 μm. (E, F) Wound-healing assays performed in H1975 (E) and PC9 (F) cells under the same transfection conditions. Representative images at 0 h and 24 h are shown. Quantitative analysis of wound width was calculated as the percentage of the initial gap width. Scale bar, 100 μm. Data are presented as mean ± SD from three independent biological replicates. Statistical analysis was performed using one-way ANOVA followed by appropriate post hoc tests. ns, not significant, *P < 0.05, **P < 0.01, ***P < 0.001.

To further evaluate whether SHC1 is involved in RP3-340N1.2-associated migratory and invasive phenotypes, dependency assays were performed in H1975 and PC9 cells. Transwell assays showed that RP3-340N1.2 overexpression was associated with an increased number of migrated cells compared with the control group, whereas simultaneous SHC1 knockdown markedly reduced this effect (Fig 9A-D). The number of migrated cells in the oe-RP3-340N1.2 + si-SHC1 group was comparable to or lower than that observed in the control group, suggesting that SHC1 depletion can counteract the migration-associated changes linked to RP3-340N1.2 overexpression.

Similarly, wound-healing assays demonstrated that RP3-340N1.2 overexpression was accompanied by accelerated wound closure at 24 h relative to the control condition (Fig 9E,F). This tendency was substantially weakened following co-transfection with si-SHC1. Quantitative analysis further supported that SHC1 knockdown reduced the RP3-340N1.2-associated alteration in cell motility.

Collectively, these findings suggest that SHC1 contributes to the migratory and invasion-related phenotypes associated with RP3-340N1.2 overexpression and may function as an important downstream component within this regulatory framework.

### Schematic summary of the RP3-340N1.2-associated regulatory network in LUAD

**Fig 10:**
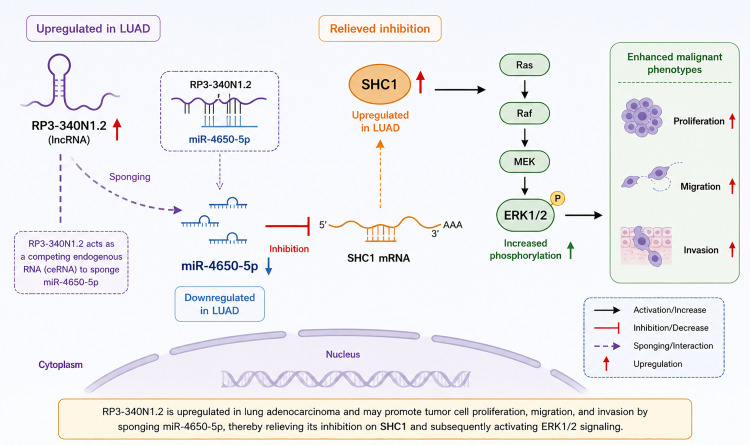
Proposed regulatory model of RP3-340N1.2 in lung adenocarcinoma. Schematic illustration of the potential regulatory framework involving RP3-340N1.2 in LUAD cells. RP3-340N1.2 is shown as upregulated and may interact with hsa-miR-4650-5p, thereby influencing its availability for target regulation. miR-4650-5p is associated with SHC1 expression, which is linked to ERK1/2 phosphorylation. Changes in this signaling context are accompanied by alterations in cellular behaviors, including proliferation, migration, and invasion. The model summarizes the observed associations and does not imply a strictly linear or exclusive regulatory relationship.

Based on the above findings, a schematic model was constructed to summarize the potential regulatory framework of RP3-340N1.2 in lung adenocarcinoma. As illustrated, RP3-340N1.2 is upregulated in LUAD and may interact with hsa-miR-4650-5p through sequence complementarity, thereby influencing the availability of miR-4650-5p for downstream target regulation. In this context, miR-4650-5p is associated with the modulation of SHC1 expression, which is linked to changes in ERK1/2 phosphorylation status. Consistent with the experimental observations, alterations in RP3-340N1.2 and miR-4650-5p expression were accompanied by corresponding variations in SHC1 protein levels and ERK signaling activity. Furthermore, functional assays suggested that these molecular changes are associated with differences in cellular behaviors, including proliferation, migration, and invasion. Taken together, this model provides an integrated view of the RP3-340N1.2/miR-4650-5p/SHC1-associated regulatory framework in LUAD, while acknowledging that additional regulatory mechanisms may also be involved (Fig 10).

## Discussion

In the present study, we investigated the expression pattern and functional relevance of the lncRNA RP3-340N1.2 in lung adenocarcinoma. Public transcriptomic analyses showed that RP3-340N1.2 was upregulated in LUAD tissues. This finding was further supported by in vitro validation in LUAD cell lines. Functional assays further suggested that RP3-340N1.2 may be involved in regulating malignant phenotypes of LUAD cells. These findings provide initial evidence that RP3-340N1.2 is associated with LUAD progression and may participate in tumor-related regulatory networks.

LncRNAs have increasingly been recognized as important modulators of gene expression in cancer, particularly through post-transcriptional mechanisms involving microRNAs. One commonly described mechanism is the competing endogenous RNA model. In this model, lncRNAs interact with miRNAs through sequence complementarity and may influence downstream target regulation(10, 15). In this study, bioinformatic prediction using the LncBase Experimental v3 database identified hsa-miR-4650-5p as a candidate miRNA potentially interacting with RP3-340N1.2. Subsequent luciferase reporter assays and RNA immunoprecipitation experiments supported a direct interaction between RP3-340N1.2 and miR-4650-5p. Importantly, mutation of the predicted binding sites attenuated this interaction, indicating that the observed association is sequence-dependent rather than nonspecific.

Studies have shown that the novel miRNA miR-4650-5p can regulate neural differentiation in SH-SY5Y cells through the Wnt/MAPK pathway [28]. Although this observation was obtained in a neural differentiation model rather than in cancer cells, it suggests that miR-4650-5p may be associated with MAPK-related signaling contexts. In the present study, we found that miR-4650-5p was linked to regulation of SHC1, an adaptor protein involved in receptor tyrosine kinase-mediated signal transduction and downstream ERK/MAPK activation. Therefore, our findings may represent a distinct cancer-related context in which miR-4650-5p participates in ERK/MAPK-associated regulation through SHC1. Nevertheless, the upstream signaling environment in LUAD is likely different from the Wnt/MAPK-related process reported in neuronal differentiation, and additional studies are needed to clarify whether these pathways are mechanistically interconnected [29]. In other areas, miR-4650-5p has not been investigated. RP3-340N1.2 appeared to influence miR-4650-5p–associated regulatory effects without necessarily suppressing miR-4650-5p transcription. This is consistent with the ceRNA model, in which lncRNAs mainly act through competitive binding rather than direct regulation of miRNA abundance.

Further analysis identified SHC1 as a potential downstream effector within this regulatory framework. SHC1 is an adaptor protein involved in signal transduction downstream of receptor tyrosine kinases. It has also been implicated in oncogenic pathways related to cell proliferation and survival [30–32]. Our data indicated that modulation of RP3-340N1.2 and miR-4650-5p influenced SHC1 expression and downstream ERK signaling activity. These findings suggest a functional link between the RP3-340N1.2/miR-4650-5p axis and SHC1-associated pathways. However, the involvement of additional targets or parallel regulatory mechanisms cannot be excluded.

Recent advances in ncRNA research have highlighted their potential translational relevance in cancer diagnosis, prognostic stratification, and targeted therapy. Increasing interest has focused on the possibility of exploiting lncRNA-associated regulatory networks as biomarkers or therapeutic targets in precision oncology [33,34]. Although the present study remains exploratory, the findings provide preliminary evidence supporting the potential involvement of RP3-340N1.2 in LUAD-associated regulatory pathways and may contribute to future investigations of ncRNA-based therapeutic strategies.

Several limitations of this study should be acknowledged. First, the functional analyses were primarily conducted in vitro using LUAD cell lines, and the biological relevance of RP3-340N1.2 in vivo remains to be further explored. Second, while SHC1 was examined as a representative downstream target, miR-4650-5p may regulate multiple genes, and the broader regulatory landscape of this miRNA warrants additional investigation. In addition, the xenograft experiments were conducted with a relatively small number of animals per group. Although statistically significant differences in tumor growth were observed, the limited sample size may reduce the generalizability of the in vivo findings. Future studies using larger animal cohorts and independent validation experiments would help further strengthen the robustness of the observed effects. Finally, RP3-340N1.2-associated signaling may extend beyond the pathways evaluated in this study. Future studies using complementary approaches may provide a more comprehensive understanding.

In summary, this study provides evidence that RP3-340N1.2 is upregulated in LUAD and is associated with malignant cellular behaviors. Our findings suggest that RP3-340N1.2 may function, at least in part, through interaction with miR-4650-5p and modulation of downstream signaling components. These results add to current knowledge of lncRNA-mediated regulation in LUAD. They also highlight RP3-340N1.2 as a potential regulatory molecule worthy of further investigation.

## Supporting information

S1 Table Summary of miR-4650-5p target prediction results from TargetScan, miRDB, and miRWalk, including the numbers of predicted target genes, overlapping candidate genes, and the complete list of genes identified in the three-database intersection. (XLSX)

S1 Fig Original uncropped and unadjusted western blot images underlying the results presented in Figs 7–9. (PDF)

S1 Data Numerical data underlying all graphs and quantitative analyses reported in this study. (XLSX)

S1 File Humane-endpoints-checklist. (DOCX)
