# Supplementary figures and images for: Characterization of a novel lncRNA RP3-340N1.2 and its association with a miR-4650-5p/SHC1-related regulatory network in lung adenocarcinoma
Source: PLoS One. 2026 Jul 16;21(7):e0353744. doi: 10.1371/journal.pone.0353744 (PMC13375034; doi:10.1371/journal.pone.0353744)

SHC1-1

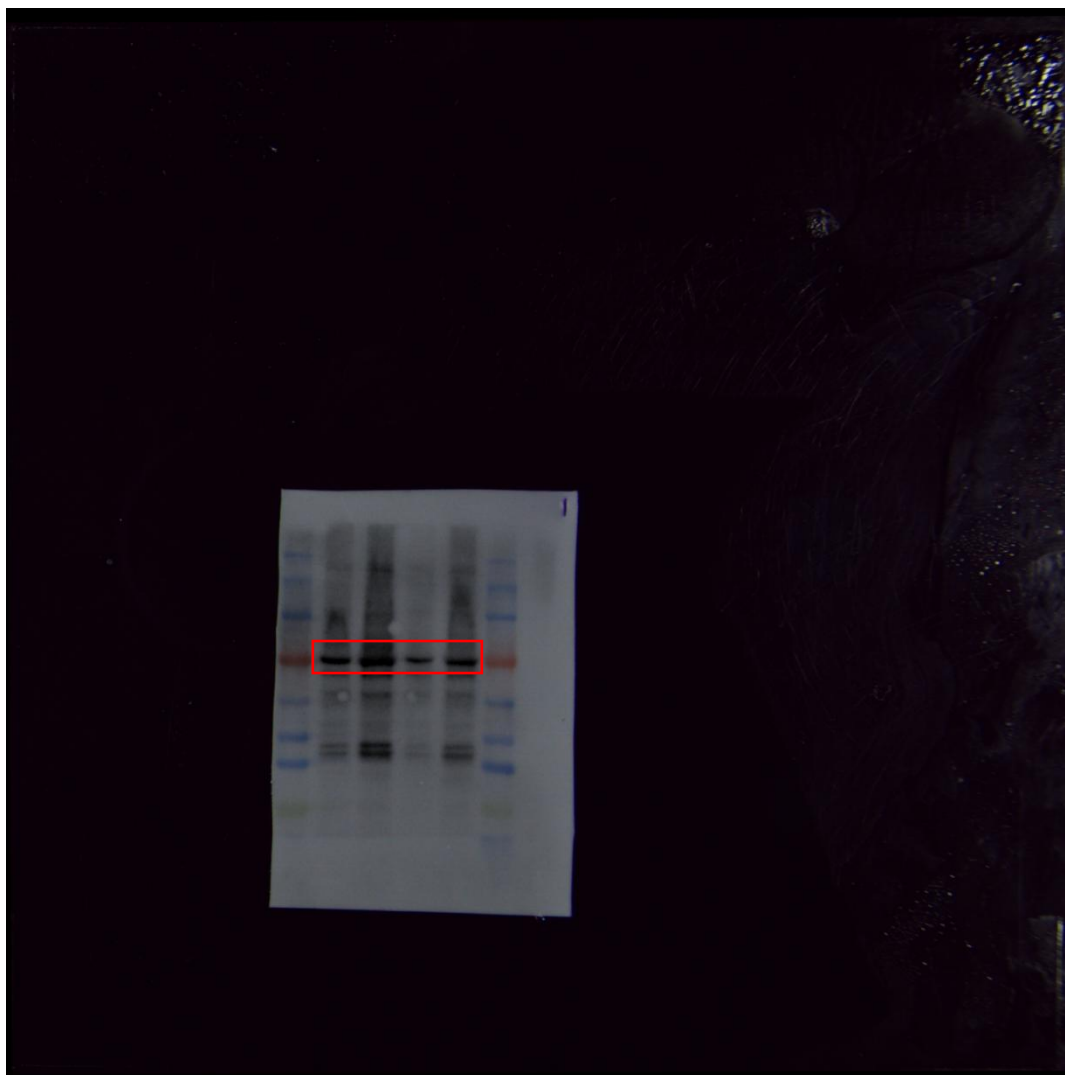

SHC1-2

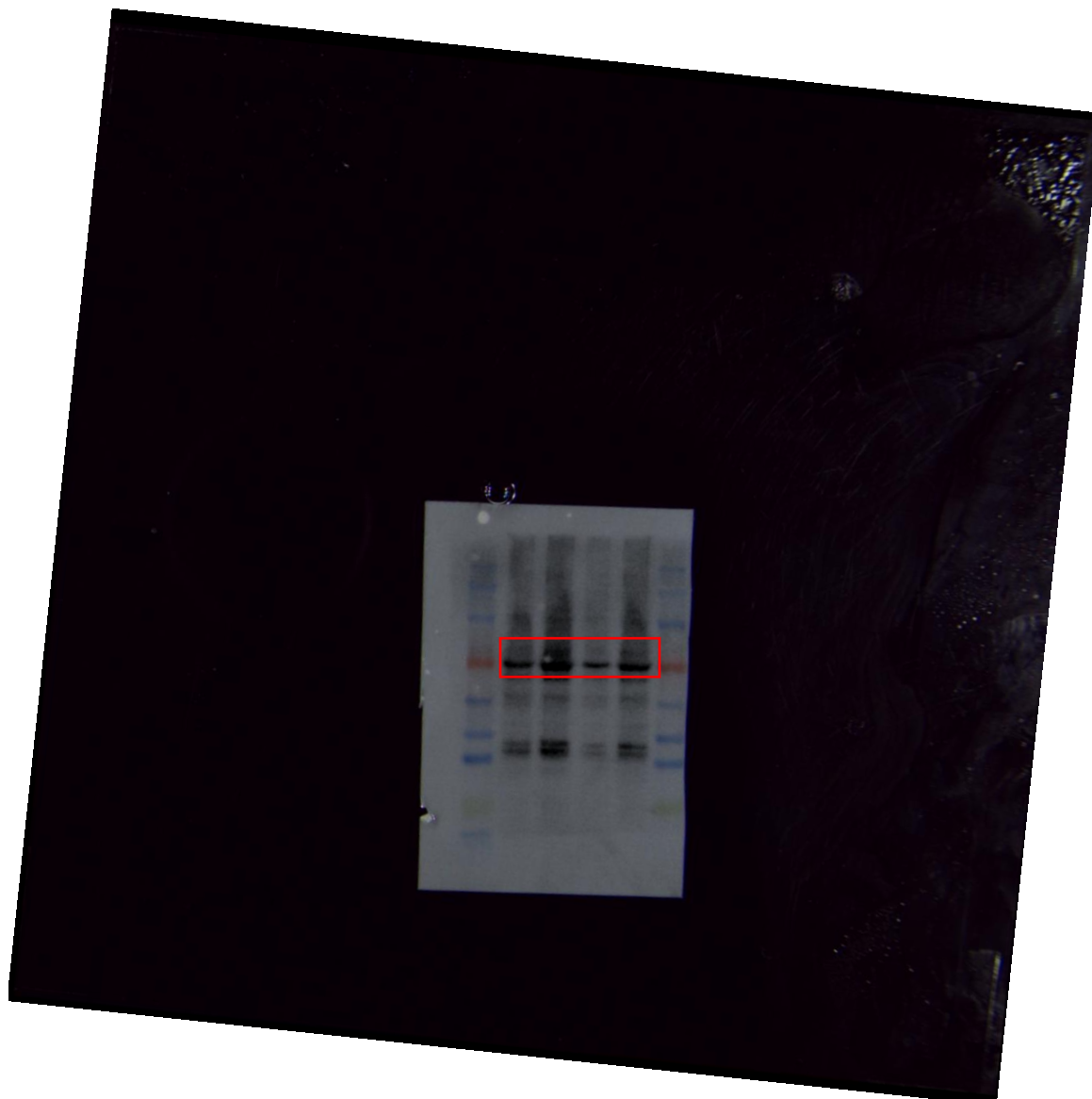

Beta-actin-1

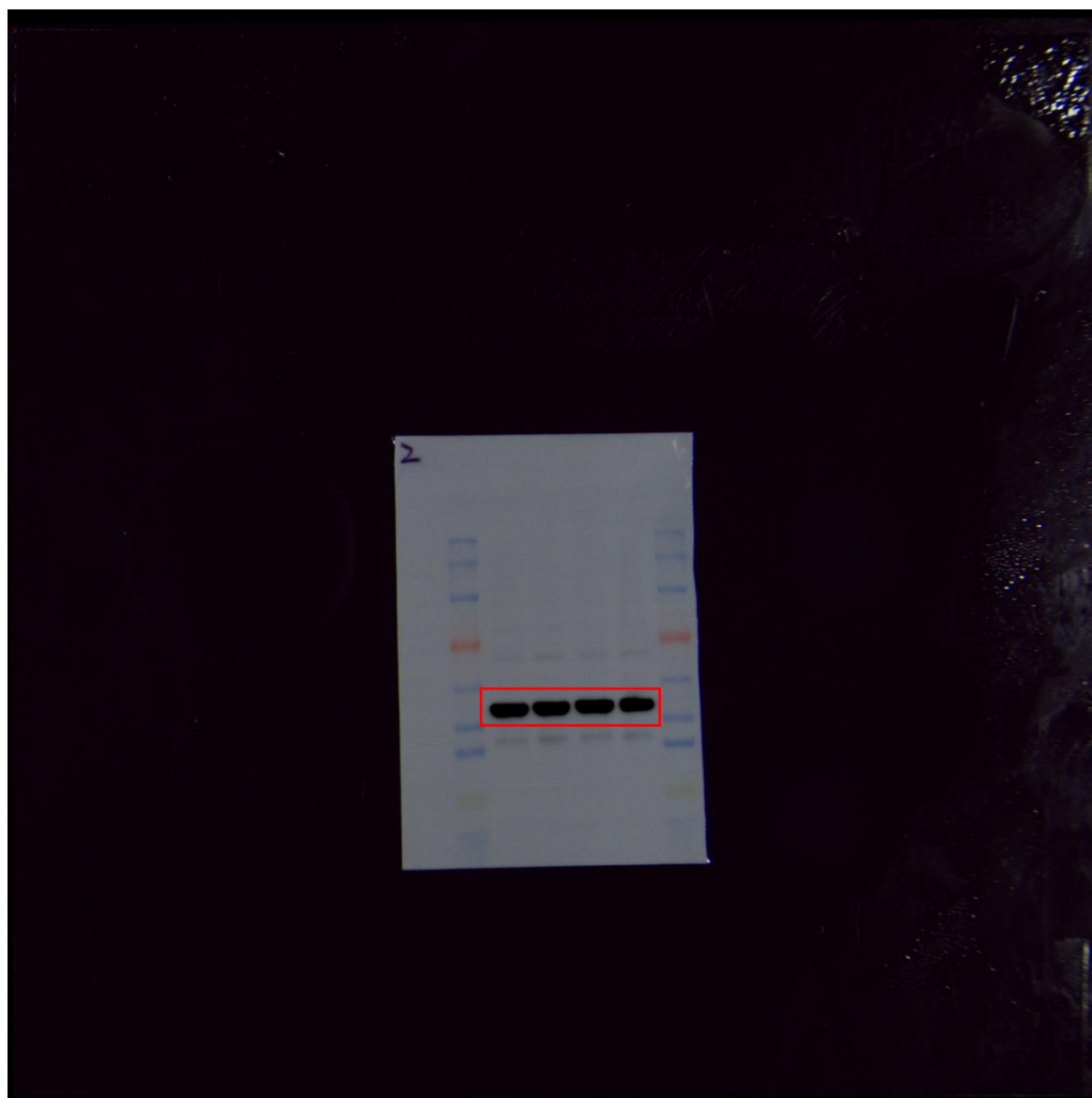

Beta-actin-2

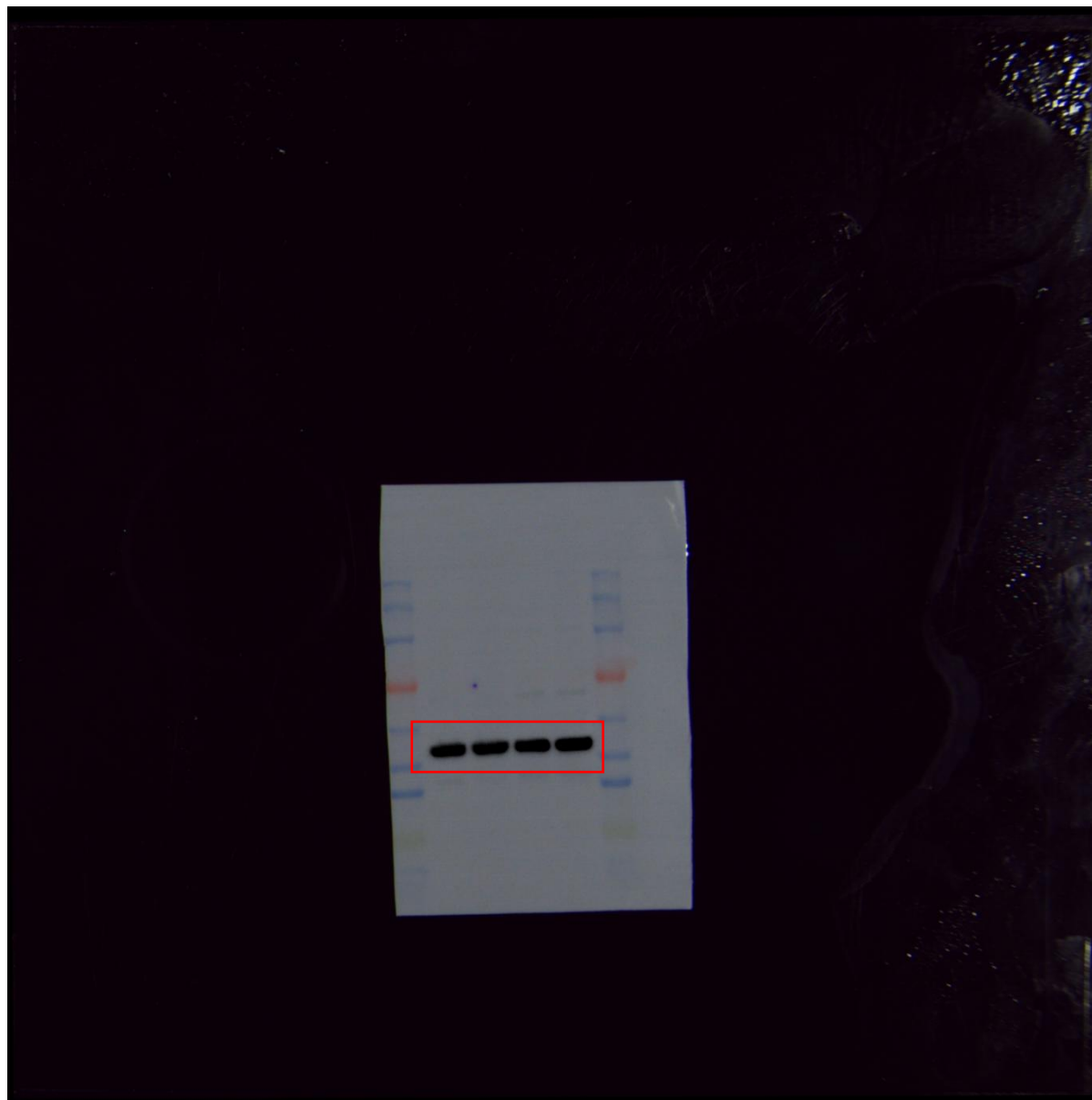

SHC1-3

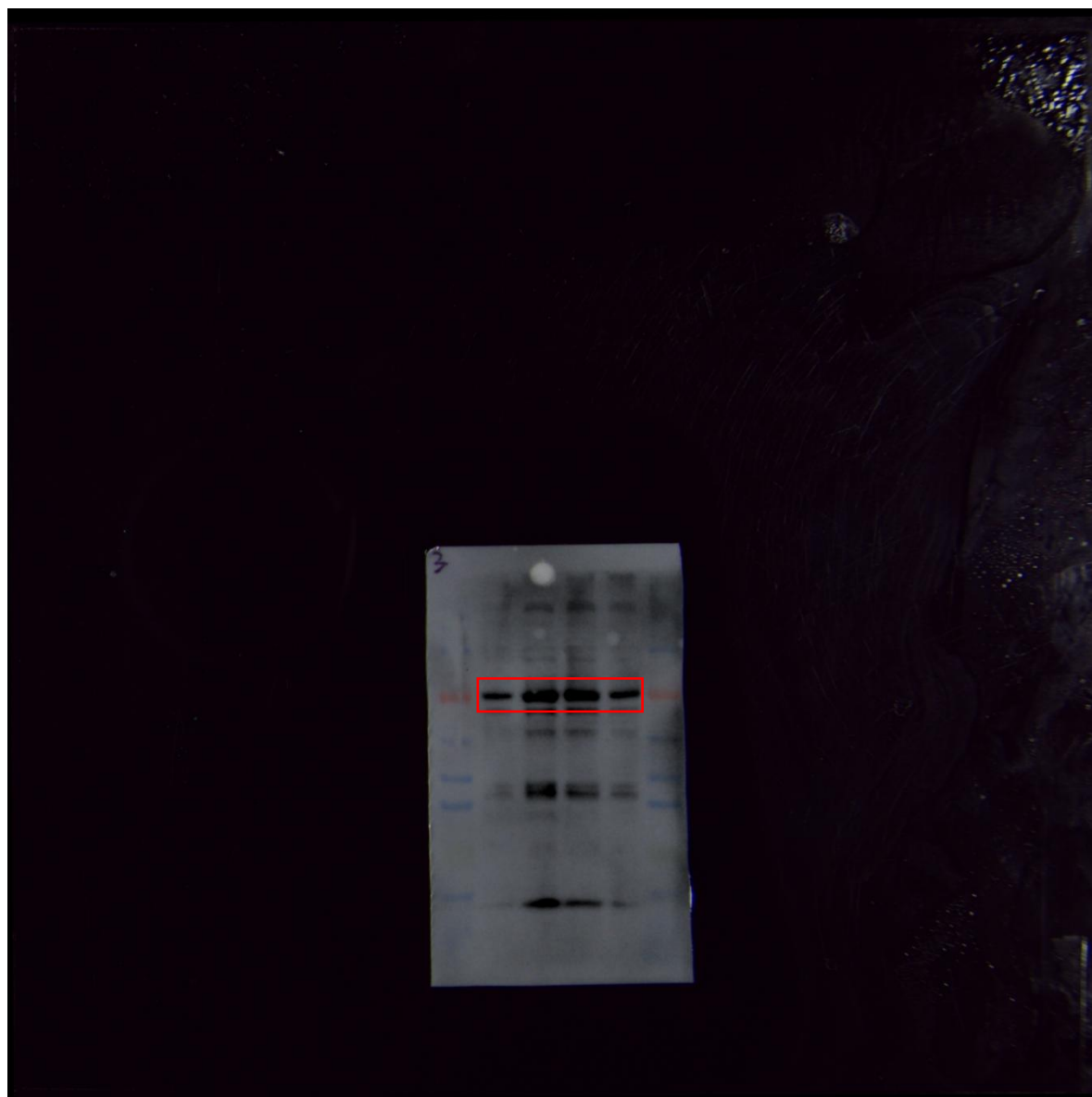

SHC1-4

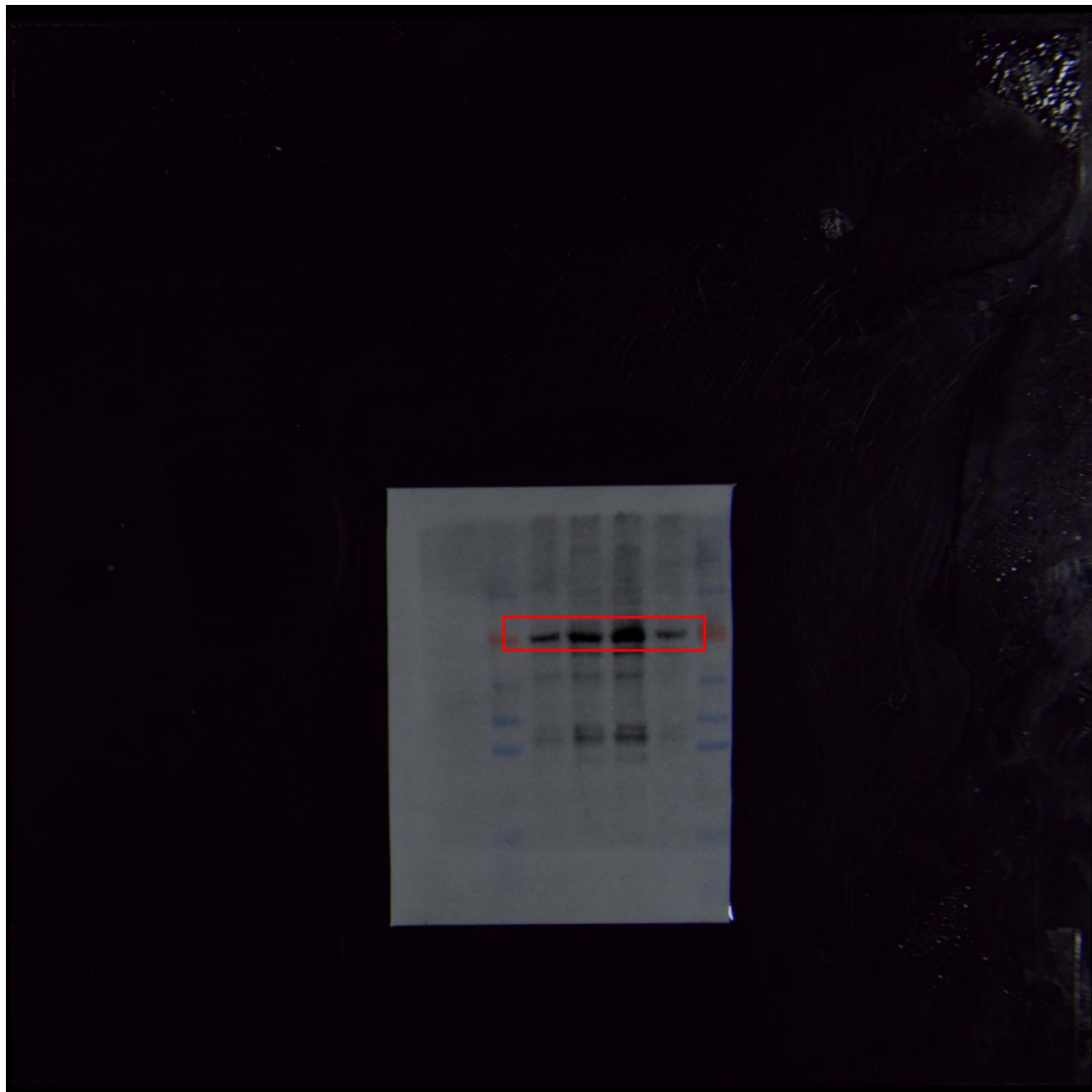

P-ERK1/2-1

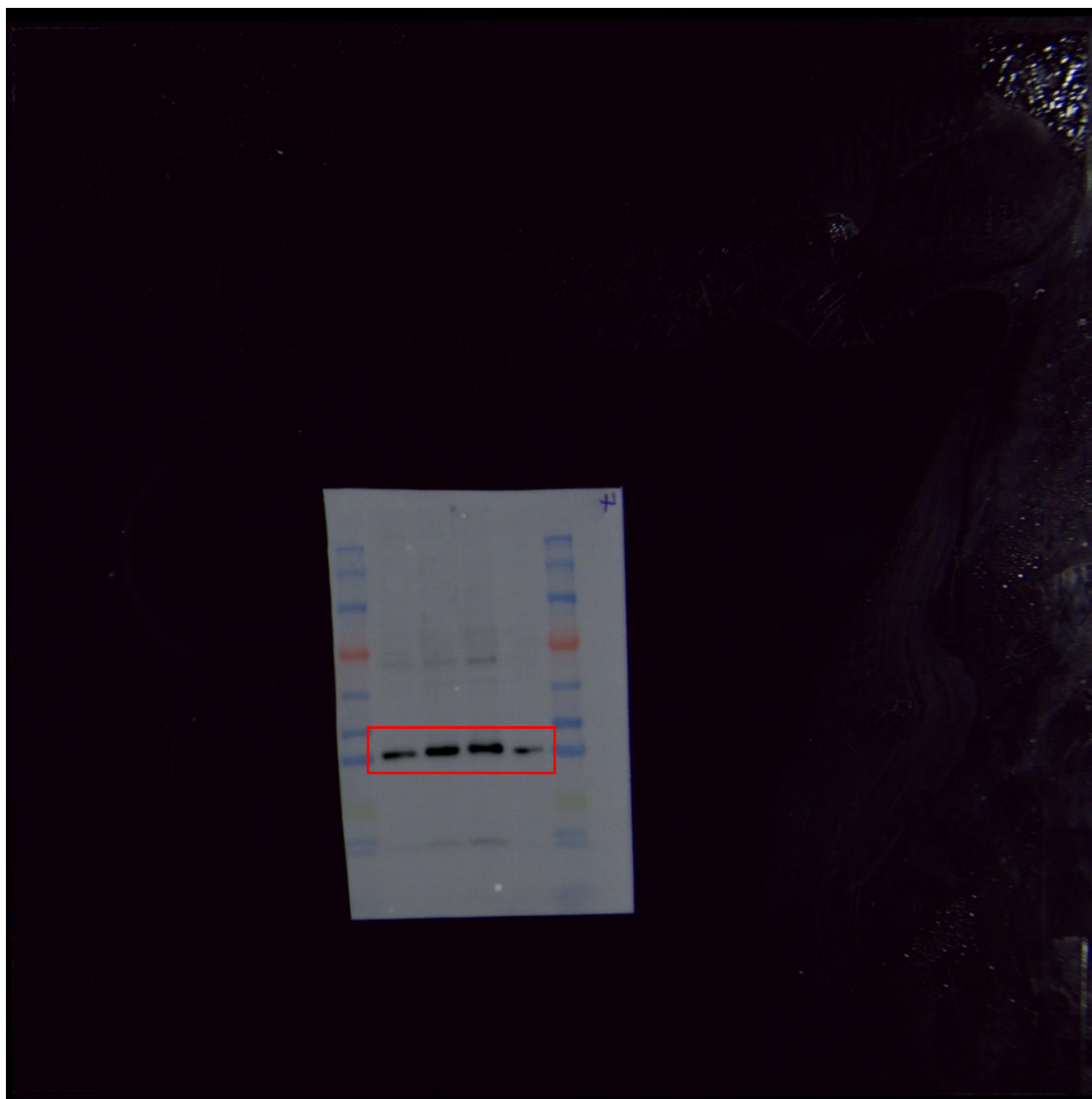

p-ERK1/2-2

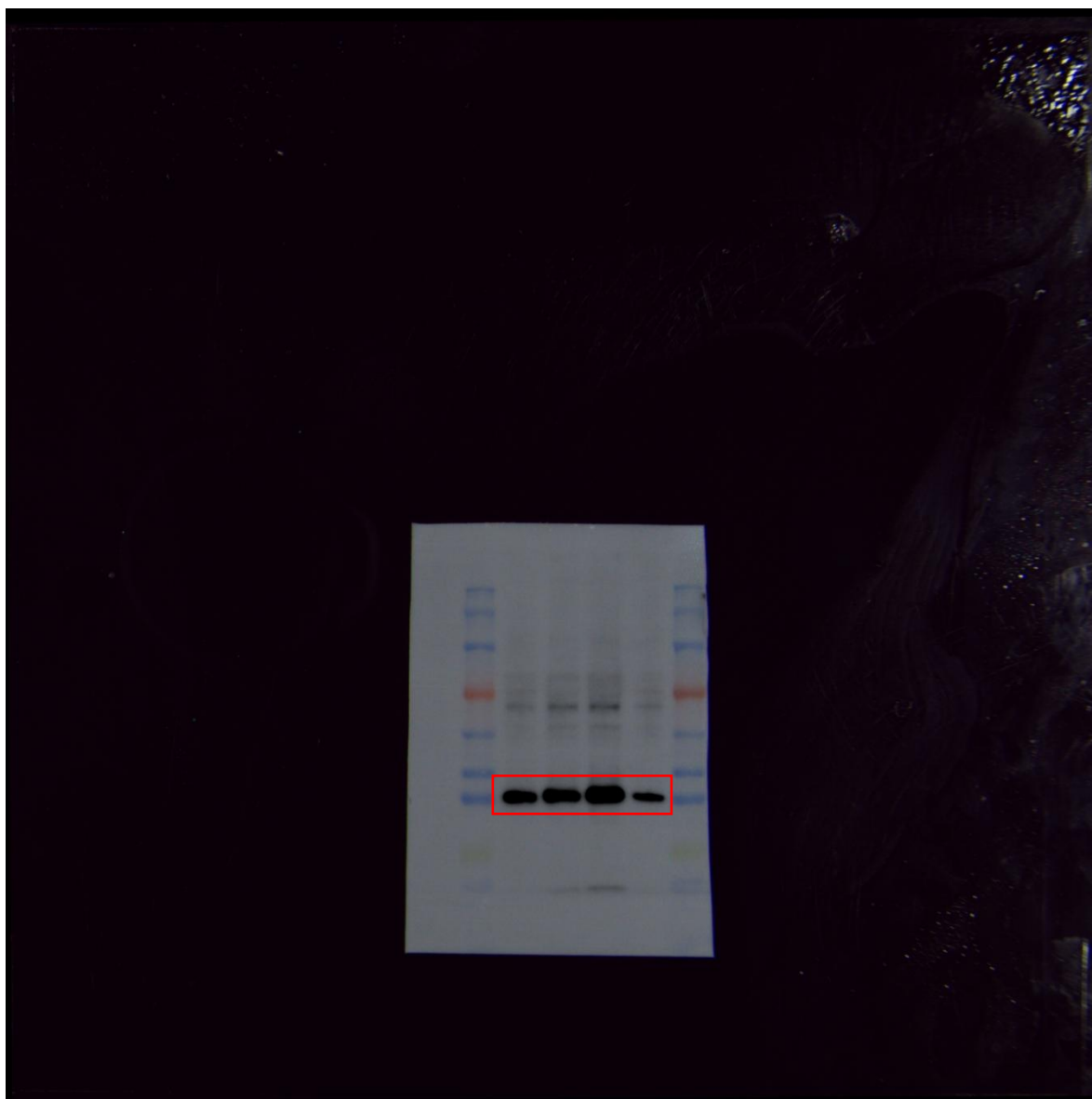

ERK1/2-1

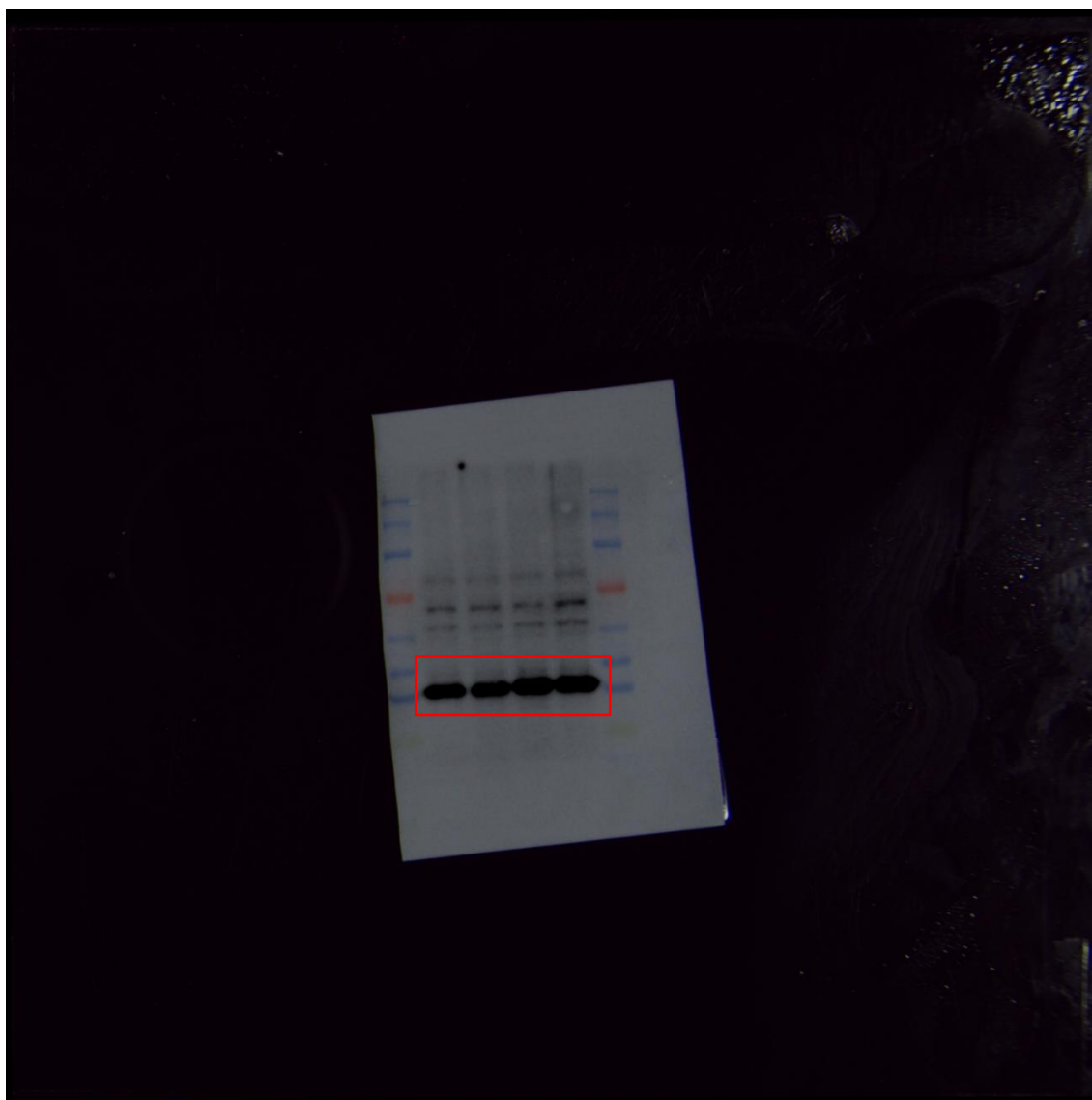

ERK1/2-2

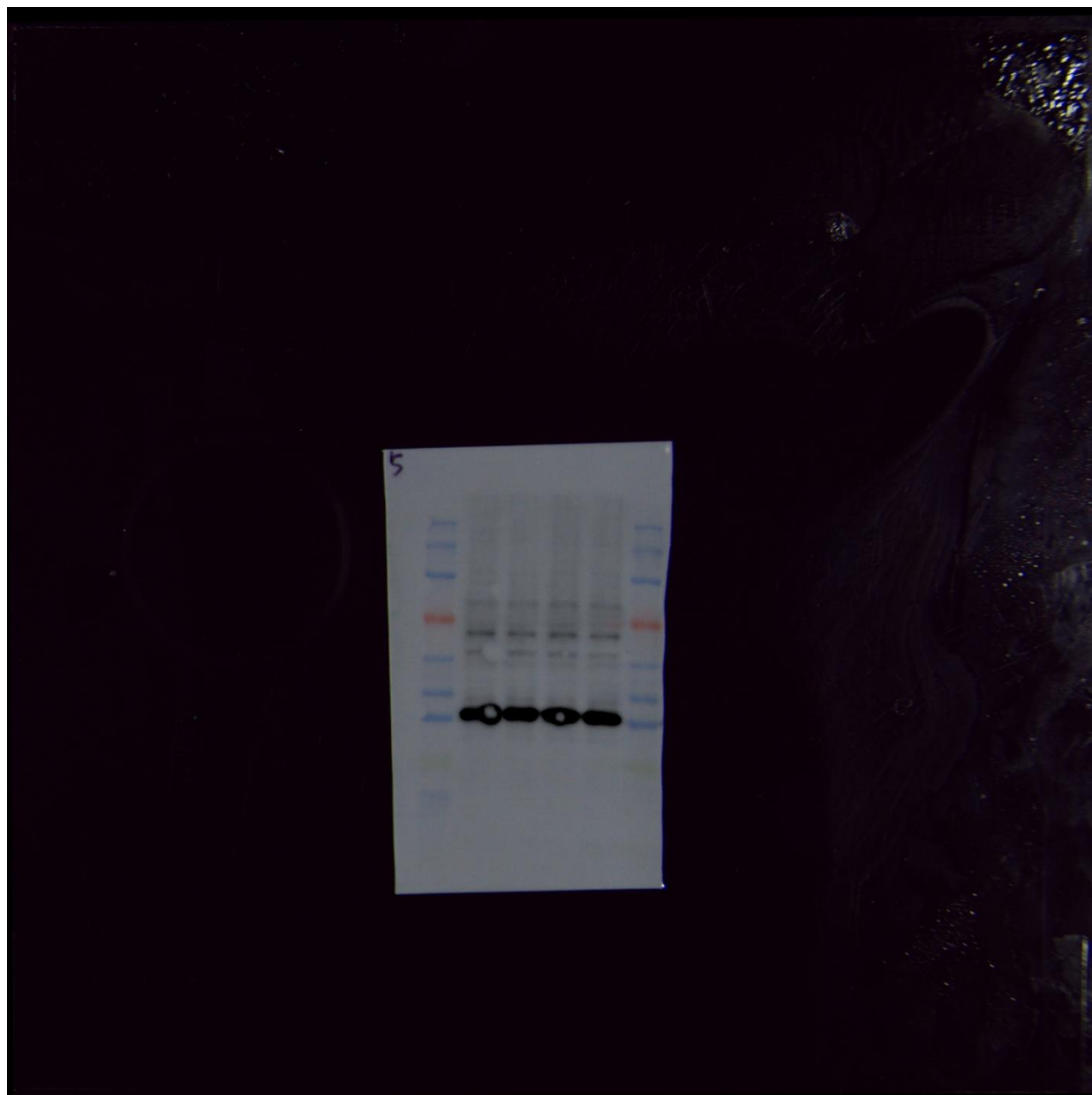

Beta-actin-3

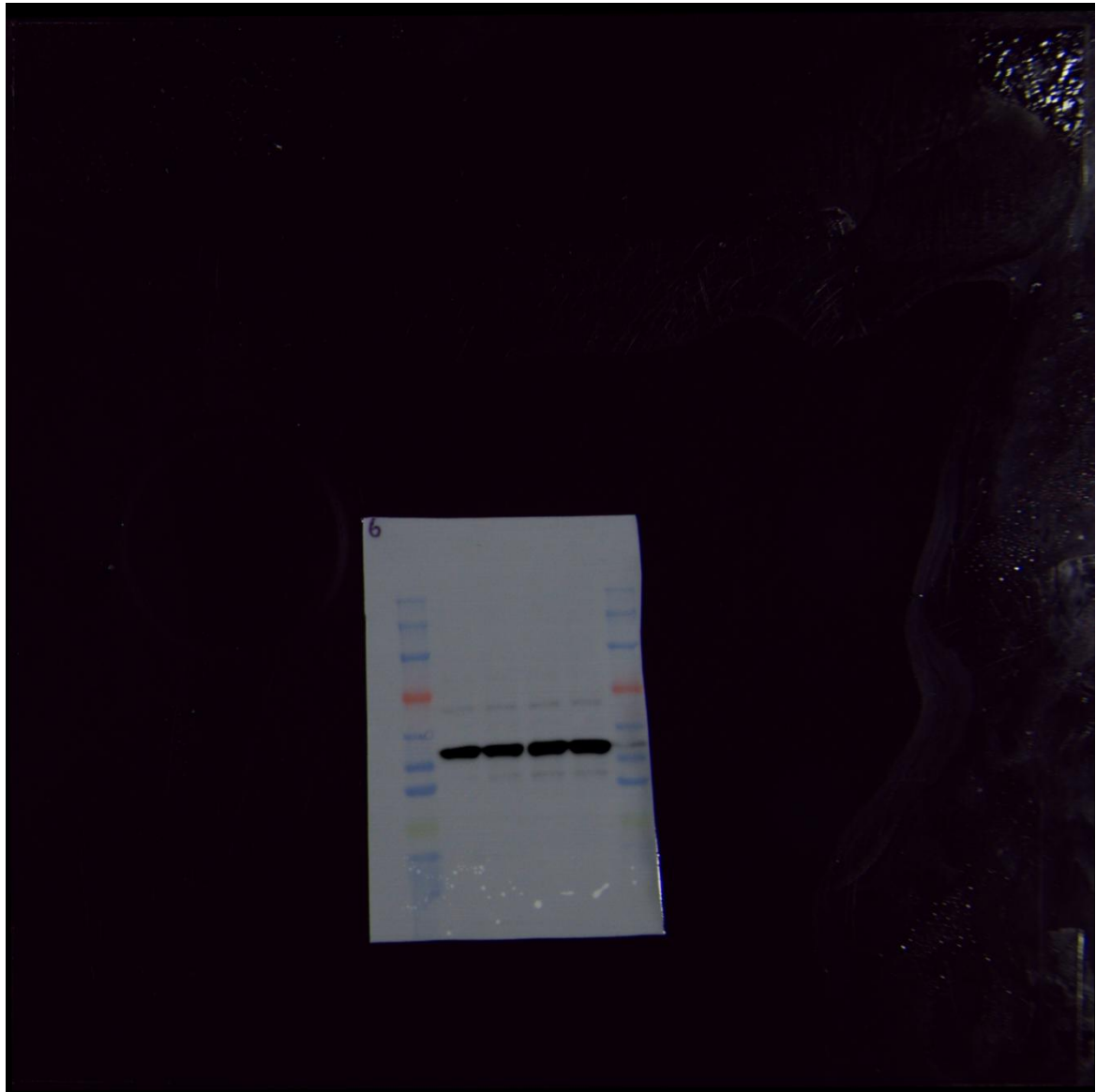

Beta-actin-4

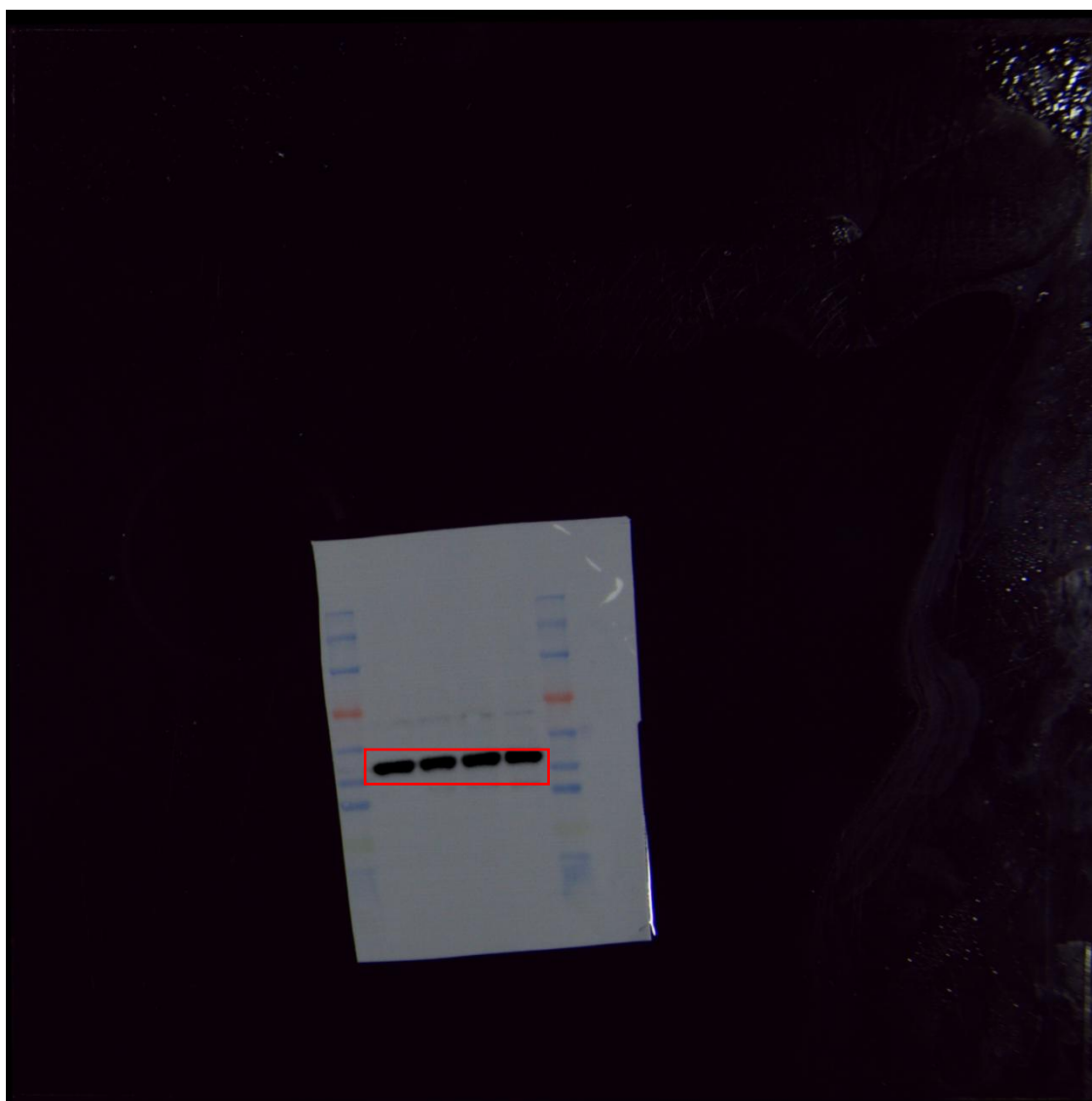

Marker

WJ103

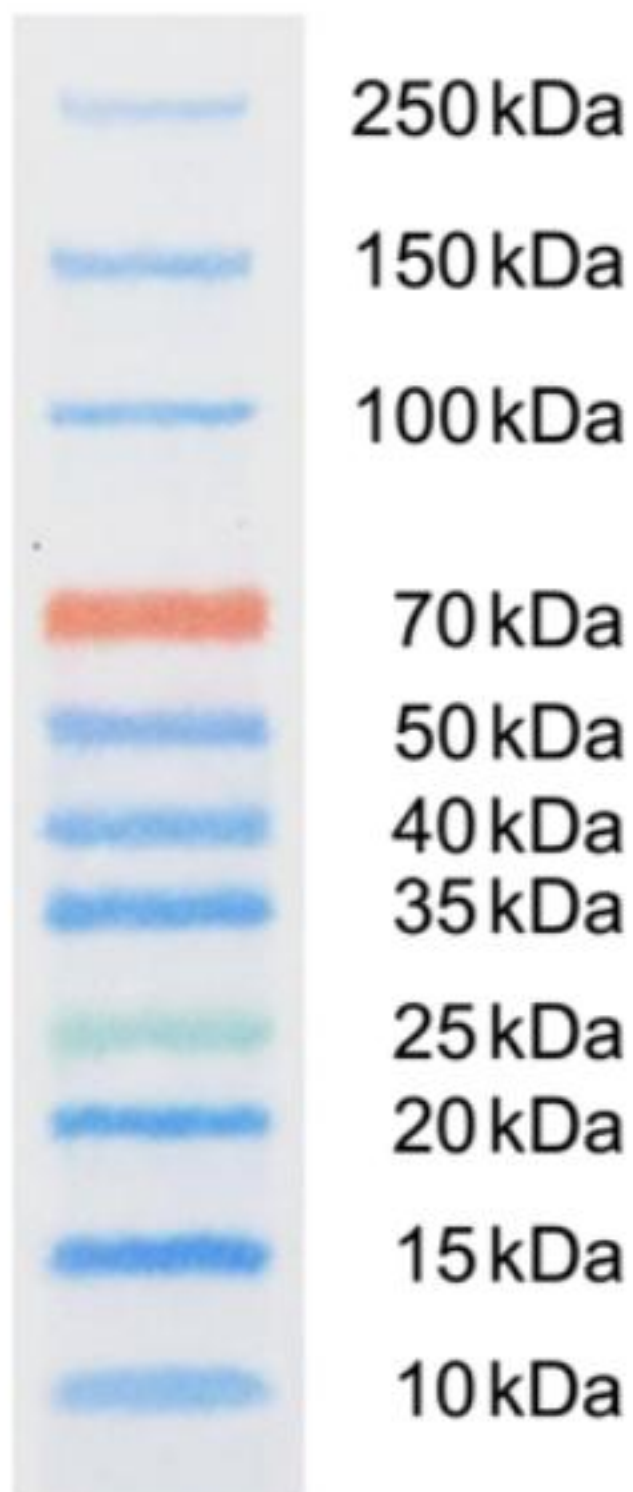

Supplement: S1 Fig — (PDF) [file pone.0353744.s002.pdf]
